# Integrating HIV services and other health services: A systematic review and meta-analysis

**DOI:** 10.1371/journal.pmed.1003836

**Published:** 2021-11-09

**Authors:** Caroline A. Bulstra, Jan A. C. Hontelez, Moritz Otto, Anna Stepanova, Erik Lamontagne, Anna Yakusik, Wafaa M. El-Sadr, Tsitsi Apollo, Miriam Rabkin, UNAIDS Expert Group on Integration, Rifat Atun, Till Bärnighausen

**Affiliations:** 1 Heidelberg Institute of Global Health, Heidelberg University Medical Center, Heidelberg, Germany; 2 Department of Public Health, Erasmus University Medical Center, Rotterdam, The Netherlands; 3 Joint United Nations Programme on HIV/AIDS, Geneva, Switzerland; 4 Aix-Marseille School of Economics, CNRS, EHESS, Centrale Marseille, Aix-Marseille University, Les Milles, France; 5 ICAP, Columbia University, New York, New York, United States of America; 6 Ministry of Health and Child Care, Harare, Zimbabwe; 7 Harvard Center for Population and Development Studies, Harvard University, Cambridge, Massachusetts, United States of America; 8 Africa Health Research Institute, KwaZulu-Natal, South Africa; St. Paul’s Hospital, CANADA

## Abstract

**Background:**

Integration of HIV services with other health services has been proposed as an important strategy to boost the sustainability of the global HIV response. We conducted a systematic and comprehensive synthesis of the existing scientific evidence on the impact of service integration on the HIV care cascade, health outcomes, and cost-effectiveness.

**Methods and findings:**

We reviewed the global quantitative empirical evidence on integration published between 1 January 2010 and 10 September 2021. We included experimental and observational studies that featured both an integration intervention and a comparator in our review. Of the 7,118 unique peer-reviewed English-language studies that our search algorithm identified, 114 met all of our selection criteria for data extraction. Most of the studies (90) were conducted in sub-Saharan Africa, primarily in East Africa (55) and Southern Africa (24). The most common forms of integration were (i) HIV testing and counselling added to non-HIV services and (ii) non-HIV services added to antiretroviral therapy (ART). The most commonly integrated non-HIV services were maternal and child healthcare, tuberculosis testing and treatment, primary healthcare, family planning, and sexual and reproductive health services. Values for HIV care cascade outcomes tended to be better in integrated services: uptake of HIV testing and counselling (pooled risk ratio [RR] across 37 studies: 1.67 [95% CI 1.41–1.99], *p <* 0.001), ART initiation coverage (pooled RR across 19 studies: 1.42 [95% CI 1.16–1.75], *p =* 0.002), time until ART initiation (pooled RR across 5 studies: 0.45 [95% CI 0.20–1.00], *p =* 0.050), retention in HIV care (pooled RR across 19 studies: 1.68 [95% CI 1.05–2.69], *p =* 0.031), and viral suppression (pooled RR across 9 studies: 1.19 [95% CI 1.03–1.37], *p =* 0.025). Also, treatment success for non-HIV-related diseases and conditions and the uptake of non-HIV services were commonly higher in integrated services. We did not find any significant differences for the following outcomes in our meta-analyses: HIV testing yield, ART adherence, HIV-free survival among infants, and HIV and non-HIV mortality. We could not conduct meta-analyses for several outcomes (HIV infections averted, costs, and cost-effectiveness), because our systematic review did not identify sufficient poolable studies. Study limitations included possible publication bias of studies with significant or favourable findings and comparatively weak evidence from some world regions and on integration of services for key populations in the HIV response.

**Conclusions:**

Integration of HIV services and other health services tends to improve health and health systems outcomes. Despite some scientific limitations, the global evidence shows that service integration can be a valuable strategy to boost the sustainability of the HIV response and contribute to the goal of ‘ending AIDS by 2030’, while simultaneously supporting progress towards universal health coverage.

## Abstract 2

Caroline Bulstra and co-workers assess evidence on the benefits of service integration in the HIV care cascade.

## Author summary

**Why was this study done?:**

- The rapid scale-up of HIV testing and antiretroviral therapy (ART) in many countries and communities over the past 2 decades has been largely achieved with stand-alone HIV programmes.
- Increasing life expectancy and the side effects of ART are leading to more co-morbidities among people living with HIV, suggesting that ART programmes that also offer other treatments could improve both healthcare effectiveness and the patient experience.
- Other reasons for integration of services include the hope that joint delivery of services will increase coverage and reduce costs.
- The global evidence on integration of HIV services and other health services, to our knowledge, has never been synthesised, and it is thus unclear what the empirical effects of integration are.

**What did the researchers do and find?:**

- We conducted a systematic review and meta-analysis to synthesise the results of integrating HIV services and other health services for HIV care cascade outcomes (testing, linkage to care, treatment initiation, treatment adherence, retention, and viral suppression), HIV health outcomes (new infections and mortality), non-HIV health outcomes, and costs and cost-effectiveness.
- In most of the 114 studies that our systematic review identified most outcomes were better in integrated compared to separate services.

**What do these findings mean?:**

- Integration of HIV services and other health services tends to improve health and health systems outcomes.
- The success of integration strategies is highly context-specific, and more evidence is needed on integration in specific geographical areas and for key populations in the HIV response.
- Despite such limitations, our systematic review and meta-analysis support the case for integration as a valuable and viable strategy to boost the sustainability of the HIV response and contribute to the goal of ‘ending AIDS by 2030’, while simultaneously supporting progress towards universal health coverage.

## Introduction

Ambitious goals guide the global HIV response. The 2016 political declaration of the United Nations General Assembly on HIV and AIDS [1] reinforced the commitment of the international community to reach the ‘getting to zero’ targets: zero new HIV infections, zero AIDS-related deaths, and zero discrimination by 2030 [2,3]. The so-called fast-track commitments for HIV prevention aim to achieve access to combination prevention for 90% of all key populations in the HIV response (e.g., young women and adolescent girls, men who have sex with men, transgender people, sex workers and their clients, people who inject drugs, and prisoners) by the end of 2020 [2]. The UNAIDS ‘95-95-95 targets’ focus on near-universal and effective coverage with HIV testing and antiretroviral therapy (ART) for people living with HIV (PLHIV) [4]. Despite progress, these ambitious goals remain elusive in many countries [5,6]. Political and financial support for the global HIV response has stagnated or even declined in recent years [7,8]. At the same time, the number of PLHIV needing treatment is expected to further rise in the future [9,10]. The economic shock and health financing crisis triggered by the coronavirus disease 2019 (COVID-19) pandemic has further reduced political attention to HIV and will likely further widen the gap between the funding required and available to achieve the global HIV goals [11].

The rapid global scale-up of HIV testing, prevention, and treatment services over the past 2 decades has been largely achieved with stand-alone programmes operating separately from other health system functions [12]. At the same time, increasing life expectancy [13,14] and the side effects of ART have led to more co-morbidities among PLHIV [15], suggesting that more integrated ART programmes could improve the HIV patient’s experience and healthcare effectiveness. From the perspective of programmes that currently do not include HIV services, such as testing or treatment, integrating HIV services may lead to powerful benefits for patients, such as HIV status knowledge and needed ART.

In the broadest sense, integration is the joining of 2 or more health services that were previously separated in some way (for instance, delivered by different health workers or at different locations). The specific integration that was the topic of our systematic review and meta-analysis is the joining of health services for HIV and at least one other disease or condition [16,17].

Integration could improve or worsen aspects of health services. From the perspective of patients and clients, integration could reduce the time and inconvenience of utilising healthcare for several diseases or conditions, and thus improve the patient experience [17,18]. From the perspective of the providers and funders of care, integration could improve processes and resource allocation [19,20]. For example, integrating the delivery of different services that the same patient needs could increase access [19] and continuity of care [21,22], and improve the clinical coordination of treatments for different diseases and thus health outcomes [23–25]. Integration could also reduce the costs of services because of synergies in joint delivery [19,20]. However, it is also plausible that integration could increase costs, because joint delivery of services for multiple diseases reduces specialisation and the efficiency gains that it brings. Another plausible risk of integration is overburdening healthcare providers [12,26], especially in areas with high HIV prevalence.

We performed a systematic review and meta-analysis of existing empirical quantitative evidence on the integration of HIV services and other health services. We included all empirical quantitative studies that compared outcomes in an intervention and a comparator group, independent of how the intervention and comparator groups were assigned, defined and measured. The reason for the broad scope of study designs that we included in our systematic review is that in our fields of study—health systems, health services, and implementation research—experiments are overall rare, while observational study designs are common. We hope that our evidence synthesis will inform policy and implementation strategies aimed at improving the reach, quality, impact, and sustainability of HIV and other health services.

## Methods

### Search strategy and selection criteria

We followed the PRISMA [27] guidelines for systematic reviews and meta-analyses (S1 PRISMA Checklist). We searched Embase, Medline Ovid (the database behind PubMed), Web of Science, EconLit (ProQuest), Cochrane Central Register of Controlled Trials, and Google Scholar to identify articles published between 1 January 2010 and 28 January 2020, and manually searched the reference lists of identified studies. The search was updated on 10 September 2021 to include studies up to this date. Search strings were constructed in collaboration with a medical librarian (see S1 File for the full search strategy). We used Medical Subject Headings (MeSH) terms and ‘all fields’ terms comprising the themes healthcare integration, HIV/AIDS, utility, health, economic and healthcare quality outcome indicators, and target populations. We included all populations in our systematic review, such as the general population and PLHIV. In addition, we specifically included terms for several key populations in the HIV response in our systematic search algorithm (men who have sex with men, transgender people, sexual and gender minorities, and sex workers) to ensure that we did not miss studies of integration targeting these populations (S1 File) [5]. Database searches were restricted to studies in English.

We included full-text, peer-reviewed experimental and observational studies that provided quantitative empirical evidence on integration with both an intervention and a comparator group. We included studies on HIV care cascade outcomes (testing, linkage to care, treatment initiation, treatment adherence, retention, and viral suppression), HIV health outcomes (new infections and mortality), non-HIV health outcomes, and costs and cost-effectiveness. These outcomes align with the 5 factors of the RE-AIM Framework to evaluate the public health impact of health system interventions [28,29]: (i) reach—HIV testing and counselling; (ii) effectiveness—viral suppression and health outcomes; (iii) adoption—cost-effectiveness; (iv) implementation—linkage to care, treatment initiation, treatment retention, and adherence; and (v) maintenance—costs.

Reasons for excluding studies were (i) no integration intervention including an HIV service, (ii) integration of health system functions other than health services, (iii) no outcome of interest, (iv) no empirical evidence (e.g., editorials, perspectives, reviews, modelling studies), (v) no comparator group, (vi) non-published/non-peer-reviewed literature, (vii) study protocols, or (viii) studies whose full text was not available. All included studies reported on integration at the point of care, but integration could also have included ‘above-patient’ and ‘above-site’ levels including healthcare providers, infrastructure, resources, monitoring, evaluation, and supply chain and management [16,30]. Studies concerning all facility types were included, from hospitals to community-level services.

Three independent reviewers (CAB, MO, and AS) screened the titles and abstracts of the primary studies that our search algorithm identified, and for those records that were eligible based on our inclusion and exclusion criteria, we examined full texts to determine eligibility for data extraction. We resolved any disagreements between the independent reviewers by consensus in a discussion with another member of our author team. We did not provide the protocol online; the review was not pre-registered.

### Data analysis

Two authors (MO and AS) independently extracted the following study data: (i) general information (title, authors, year of publication, journal), (ii) study characteristics (study objectives and aims, study design, study population, study size, study time and duration), (iii) geographical location and population, (v) intervention (description, rationale for integration, degree of integration, healthcare level of integration), (v) comparator, (vi) outcomes, (vii) results, and (viii) contextual factors. We performed a quality assessment of the primary studies that our systematic search identified using the Grading of Recommendations Assessment, Development and Evaluation (GRADE) tool [31]. For studies that did not find that integration improved outcomes, we searched the journal articles for reasons for this finding.

We expressed results (i) as risk ratios (RRs) for both HIV and non-HIV care cascade and health outcomes, (ii) as number of infections averted for new HIV infections, and (iii) in 2018 US dollars for costs and the cost components of incremental cost-effectiveness ratios (ICERs). We did meta-analyses for those outcomes for which pooling of results was meaningful (i.e., for HIV and non-HIV care cascade and health outcomes), because the integration interventions studied were the same or functionally similar. We pooled results using inverse-variance weighting. We estimated the proportion of variation across studies that was due to heterogeneity of results rather than chance (*I*^2^ statistic) [32]. We also did meta-analyses for the subset of experimental studies only. All data analyses were done using R version 3.6.3 [33]. Ethical approval was not required for this study because we only used secondary data extracted from published studies.

## Results

**Fig 1:**
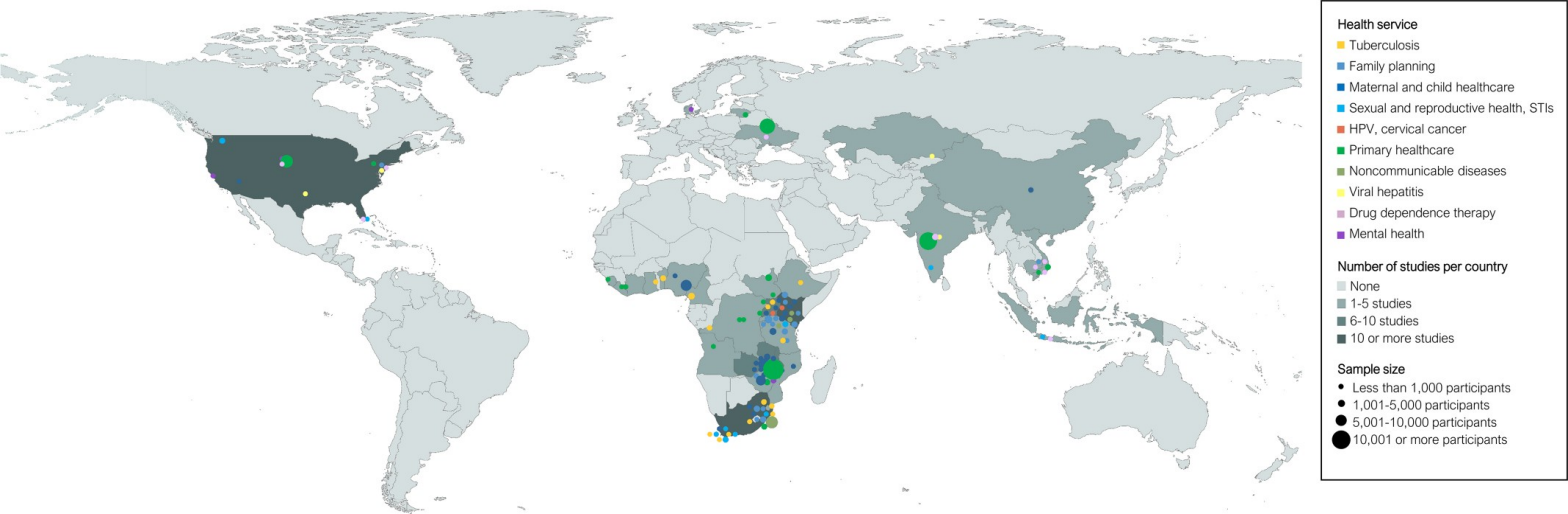
Geographical map of the included empirical studies by type of integration. Bubble colours represent the health service integration area. Bubble sizes represent the study population size. Coordinates are dispersed up to 250 kilometres to prevent overlap of data points from similar or nearby locations. Abbreviations: HPV, human papillomavirus; STI, sexually transmitted infection. Map created using ArcGIS software by ESRI. Base map source: https://www.naturalearthdata.com/downloads/10m-cultural-vectors/10m-admin-0-countries/.

Our search identified a total of 7,118 unique publications, of which 114 met the inclusion criteria (S1 Fig) [34–147]. Of the 114 included studies, 39 were experimental and 75 were observational. Fig 1 shows the geographical location and year of publication of the included studies by type of health service. Most of the studies were conducted in East Africa (55 studies) and Southern Africa (24 studies)—primarily in Kenya (20 studies), South Africa (18 studies), Zambia (8 studies), Malawi (8 studies), and Uganda (8 studies). The most common services integrated with HIV services were maternal and child health (MCH) (28 studies), tuberculosis (TB) (16 studies), family planning (16 studies), primary healthcare (14 studies), and sexual and reproductive health (SRH) or sexually transmitted infection (STI) services (13 studies). The most common intervention was integration of HIV testing and counselling into non-HIV services (46 studies), followed by integration of additional services into ART (28 studies) and integration of ART into non-HIV programmes (21 studies). Fifty studies focused on the general population, 40 studies on women or children, and 22 studies on at least 1 of the key populations in the HIV response. Study durations differed widely (S2 Fig). While the median study duration was 2 years, 28 studies were 3 years or longer, 8 studies were 5 years or longer, and the maximum duration among all studies was 8 years. S1 Table shows the distributions of study characteristics across all studies, S2 Table shows the study characteristics for each individual study, and S3 Table shows the GRADE assessment. Table 1 shows a summary of key findings.

**Table 1:** Summary of key findings.

Outcome	Relationship to global policy goals	Total number of studies (number of experimental studies)	Total study population size	Number of studies (number of experimental studies)			*I*^2^ (meta-analysis)	Pooled RR (95% CI) or mean outcome (range) based on all studies	Pooled RR (95% CI) based on experimental studies only
				Unfavourable outcome	No difference	Favourable outcome			
Uptake of HIV services	First 95 of the 95-95-95 targets	37 (11)	637,148	1 (0)	1 (0)	34 (11)	99.0%	1.67 (1.41–1.99), *p <* 0.001	1.42 (1.28–1.58), *p <* 0.001
HIV testing yield	First 95 of the 95-95-95 targets	10 (1)	461,486	3 (0)	2 (1)	5 (0)	97.7%	0.68 (0.38–1.24), *p =* 0.185	0.91 (0.61–1.36), *p =* 0.652
ART initiation	Second 95 of the 95-95-95 targets	19 (6)	271,689	2 (1)	1 (0)	16 (5)	99.8%	1.42 (1.16–1.75), *p =* 0.002	1.50 (0.97–2.33), *p =* 0.064
Time until ART initiation	Second 95 of the 95-95-95 targets	5 (1)	3,052	0 (0)	0 (0)	5 (1)	93.1%	0.45 (0.20–1.00), *p =* 0.050	0.13 (0.05–0.29), *p <* 0.001
Retention in care	Third 95 of the 95-95-95 targets	19 (7)	66,151	3 (1)	4 (2)	11 (4)	93.4%	1.68 (1.05–2.69), *p =* 0.031	1.46 (0.67–3.17), *p =* 0.282
ART adherence	Third 95 of the 95-95-95 targets	7 (3)	52,140	1 (0)	2 (2)	4 (1)	98.8%	1.13 (0.95–1.34), *p =* 0.146	1.06 (0.91–1.23), *p =* 0.245
Viral suppression	Third 95 of the 95-95-95 targets	9 (6)	24,615	0 (0)	2 (1)	6 (5)	46.1%	1.19 (1.03–1.37), *p =* 0.025	1.23 (1.00–1.51), *p =* 0.054
HIV-free survival among infants	‘Zero new infections’ of the ‘getting to zero’ strategy	5 (2)	242,196	0 (0)	3 (1)	2 (1)	99.5%	1.04 (0.98–1.11), *p =* 0.135	1.11 (1.03–1.20), *p =* 0.033
HIV infections averted	‘Zero new infections’ of the ‘getting to zero’ strategy	4 (3)	2,181	0 (0)	1 (1)	3 (3)	N/A^1^	1.16 infections averted per 100-person-years (range 0.0–3.6)	N/A
AIDS-related mortality	‘Zero AIDS-related deaths’ of the ‘getting to zero’ strategy	8 (4)	39,630	2 (1)	3 (1)	3 (2)	97.8%	0.72 (0.47–1.11), *p =* 0.118	0.99 (0.66–1.51), *p =* 0.985
Uptake of other health services	Non-HIV-related outcomes	32 (11)	278,042	2 (0)	4 (2)	24 (8)	98.4%	2.42 (1.59–3.66), *p <* 0.001	2.03 (1.31–3.15), *p =* 0.005
Treatment success for other diseases/conditions	Non-HIV-related outcomes	21 (5)	40,452	0 (0)	8 (3)	11 (2)	81.1%	1.56 (1.11–2.20), *p =* 0.014	1.64 (0.75–3.58), *p =* 0.156
Non-AIDS-related mortality	Non-HIV-related outcomes	6 (2)	25,879	1 (1)	1 (1)	4 (0)	94.7%	0.43 (0.16–1.17), *p =* 0.083	1.00 (0.01–1.62), *p =* 0.997
HIV-only costs	Costs and cost-effectiveness	6 (1)	119,830	2 (0)	0 (0)	4 (1)	N/A^1^	1.06 (range 0.59–1.92)^1^	N/A
Non-HIV costs	Costs and cost-effectiveness	2 (0)	202	0 (0)	0 (0)	2 (0)	N/A^1^	0.62 (range 0.50–0.73)^1^	N/A
HIV and non-HIV costs	Costs and cost-effectiveness	2 (1)	8,027	0 (0)	0 (0)	2 (1)	N/A^1^	0.83 (range 0.69–0.97)^1^	N/A
Costs of integrated services versus HIV-only costs	Costs and cost-effectiveness	7 (1)	132,306	N/A^2^	N/A^2^	N/A^2^	N/A^1^	2.31 (range 1.20–6.12)^1^	N/A
Cost-effectiveness	Costs and cost-effectiveness	6 (2)	142,881	0 (0)	1 (0)	5 (2)	N/A^1^	N/A	N/A

Colours indicate the outcome groups: UNAIDS 95-95-95 targets, purple; UNAIDS ‘getting to zero’ targets, blue; non-HIV-related outcomes, green; and costs and cost-effectiveness, orange.

^1^Meta-analysis not possible because of insufficient poolable data.

^2^Costs of both HIV and non-HIV services as compared to the costs of HIV services only; it was thus impossible to judge whether integration increased or reduced costs.

Abbreviations: ART, antiretroviral therapy; N/A, not applicable; RR, risk ratio.

### HIV cascade of care

**Fig 2:**
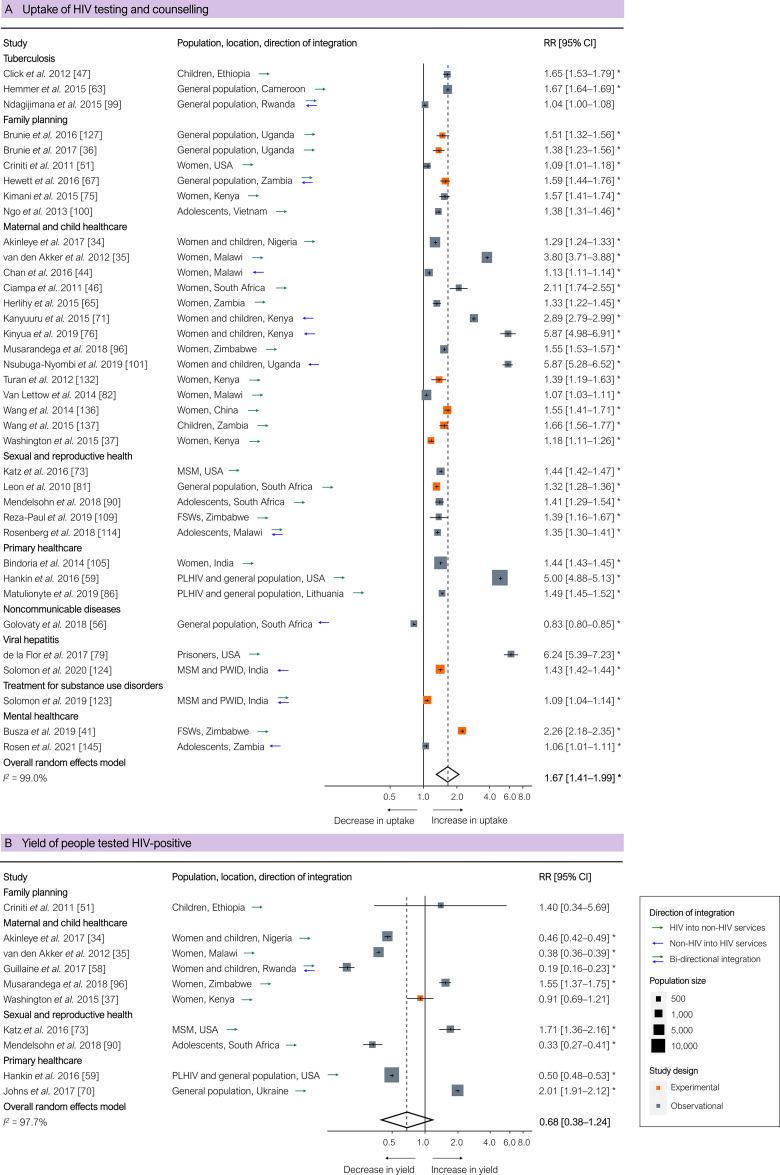
Results of integration of HIV services: Uptake of HIV testing and counselling and yield of people testing HIV-positive. (A) Uptake of HIV testing and counselling. (B) Yield of people testing HIV-positive. Outcomes are related to the ‘first 95’ of the 95-95-95 target for the HIV cascade of care. Each estimate indicates the size of the relationship between integration exposure and outcome. We measured these relationships as RRs; asterisks indicate statistically significant results. The diamond at the bottom of each panel shows the overall random-effects meta-analytical estimate. Abbreviations: CI, confidence interval; FSWs, female sex workers; MSM, men who have sex with men; PLHIV, people living with HIV; PWID, people who inject drugs; RR, risk ratio.

HIV testing and counselling uptake was significantly higher for integrated health services based on all 37 studies (pooled RR 1.67 [95% CI 1.41–1.99], *p <* 0.001; Fig 2A), including in all 11 experimental studies (pooled RR 1.42 [95% CI 1.28–1.58], *p <* 0.001). One study—evaluating integration of HIV, hypertension, and diabetes screening in rural South Africa—reported lower uptake of HIV testing, which the authors attributed to a lack of human resources and inadequate staff training [56]. Integration yielded a higher percentage of HIV-positive people in 4 out of 10 studies and a lower percentage in the remaining studies (Fig 2B).

**Fig 3:**
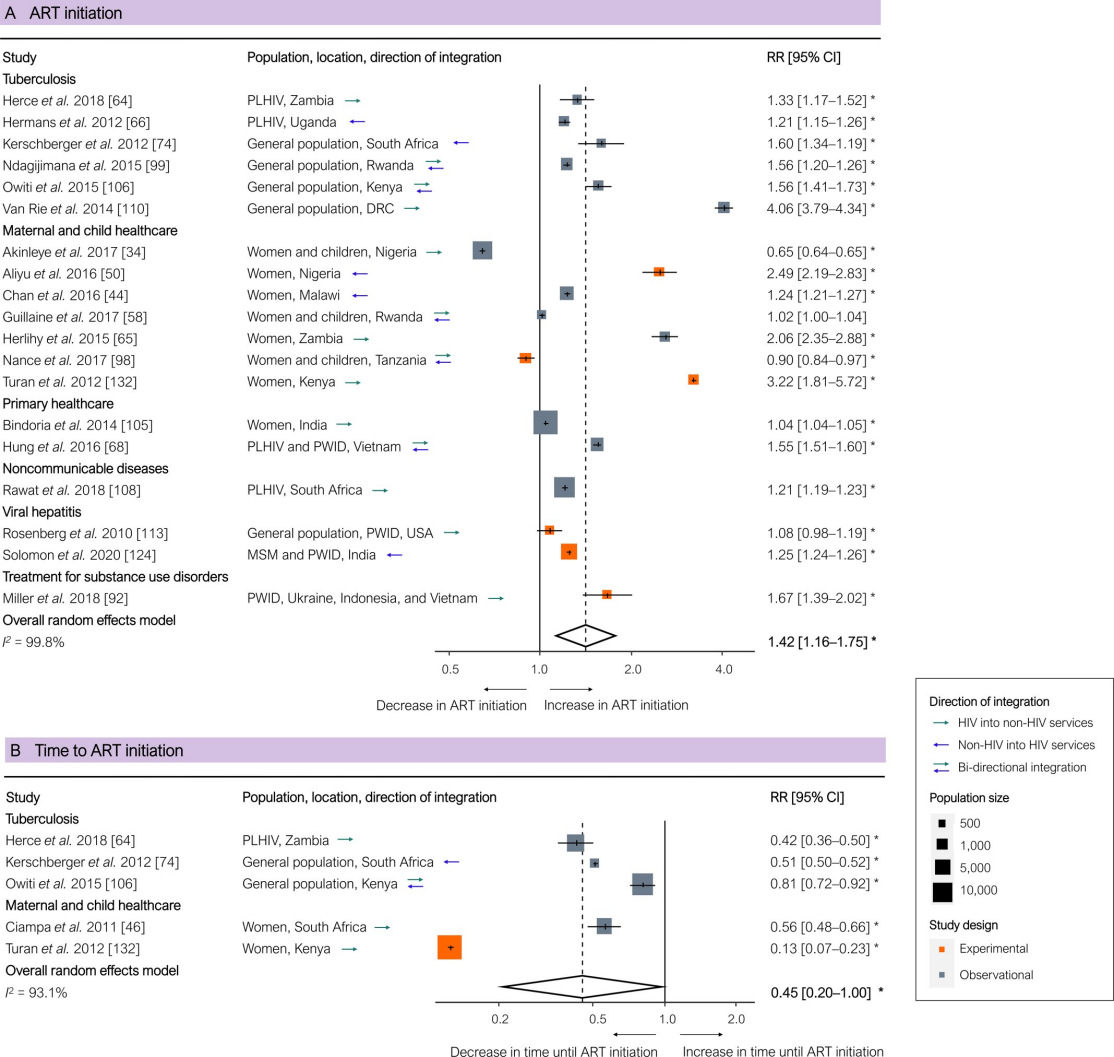
Results of integration of HIV services: ART initiation and time until ART initiation. (A) ART initiation. (B) Time until ART initiation. Outcomes are related to the ‘second 95’ of the 95-95-95 HIV cascade of care. Each estimate indicates the size of the relationship between integration exposure and outcome. We measured these relationships as RRs; asterisks indicate statistically significant results. The diamond at the bottom of each panel shows the overall random-effects meta-analytical estimate. Abbreviations: ART, antiretroviral therapy; CI, confidence interval; DRC, Democratic Republic of the Congo; MSM, men who have sex with men; PLHIV, people living with HIV; PWID, people who inject drugs; RR, risk ratio.

ART initiation was significantly higher in integrated programmes based on all 19 studies (pooled RR 1.42 [95% CI 1.16–1.75], *p =* 0.002; Fig 3A), but non-significantly higher based on the 6 experimental studies only (pooled RR 1.50 [95% CI 0.97–2.33], *p =* 0.064). Two studies that examined integration of HIV testing and counselling with MCH services reported lower initiation rates of ART and decreased prevention of mother-to-child transmission, which the authors attributed to poor linkage to HIV services beyond testing and counselling [34,98]. People initiated ART significantly faster in integrated programmes, measured in 5 studies, all of which reported on integration with TB or MCH services (pooled RR 0.45 [95% CI 0.20–1.00], *p =* 0.050; Fig 3B).

**Fig 4:**
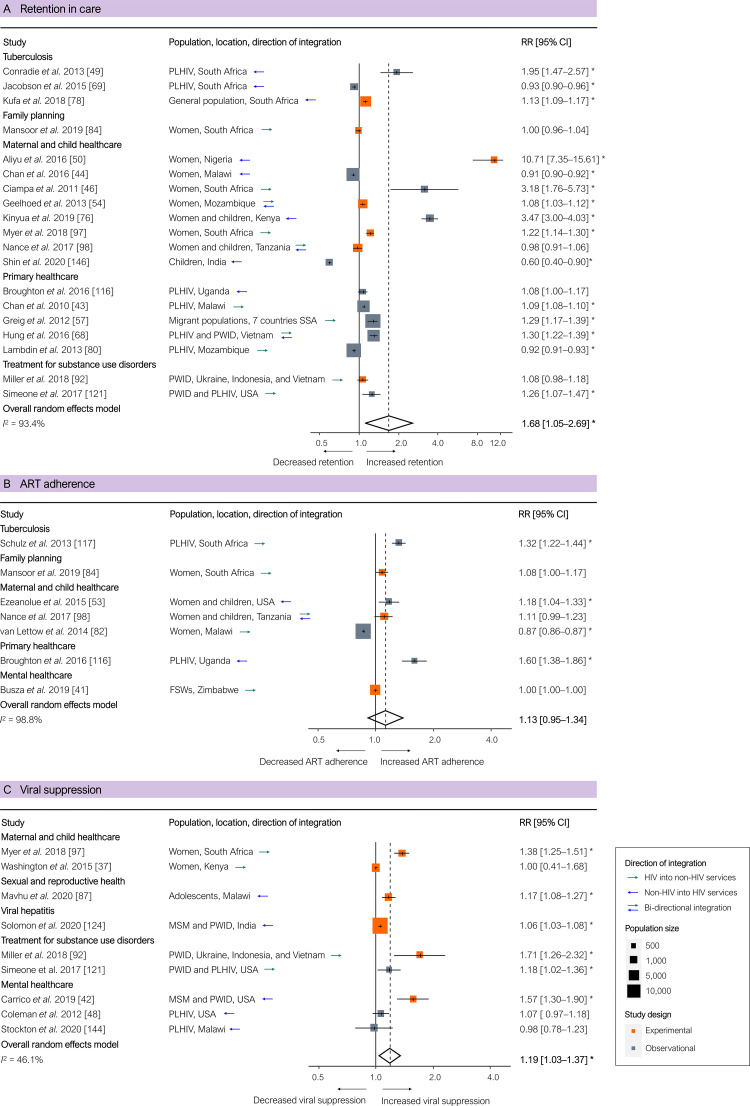
Results of integration of HIV services for PLHIV: Retention in care, ART adherence, and viral suppression of those on ART. (A) Retention in care. (B) ART adherence. (C) Viral suppression of those on ART. Outcomes are related to the ‘third 95’ of the 95-95-95 targets for the HIV cascade of care. Each estimate indicates the size of the relationship between integration exposure and outcome. We measured these relationships as RRs; asterisks indicate statistically significant results. The diamond at the bottom of each panel shows the overall random-effects meta-analytical estimate. Abbreviations: ART, antiretroviral therapy; CI, confidence interval; FSWs, female sex workers; MSM, men who have sex with men; PLHIV, people living with HIV; PWID, people who inject drugs; RR, risk ratio; SSA, sub-Saharan Africa.

Retention in care was significantly higher in integrated programmes based on all 19 studies (pooled RR 1.68 [95% CI 1.05–2.69], *p =* 0.031; Fig 4A), but non-significantly higher based on the 7 experimental studies only (pooled RR 1.46 [95% CI 0.67–3.17], *p =* 0.282). ART adherence, was non-significantly higher in integrated programmes based on all studies (pooled RR 1.13 [95% CI 0.95–1.34], *p =* 0.146; Fig 4B) and based on the 3 experimental studies only (pooled RR 1.06 [95% CI 0.91–1.23], *p =* 0.245). One study reported lower retention and adherence in integrated antenatal care and HIV services, which the researchers attributed to non-retention when women who initiated ART in antenatal care eventually had to switch from ART in antenatal care to ART in stand-alone HIV services [82]. Viral suppression, measured in 9 studies, was significantly higher in integrated programmes based on all 9 studies (pooled RR 1.19 [95% CI 1.03–1.37], *p =* 0.025; Fig 4C), and borderline significantly higher based on the 6 experimental studies only (pooled RR 1.23 [95% CI 1.00–1.51], *p =* 0.054). One study reported no significant difference in viral suppression between integrated and separate services, which the researchers attributed to the scarcity of staff to provide HIV services and insufficient adherence support in the integrated programmes [37].

### New HIV infections, HIV-related mortality, and stigma

**Fig 5:**
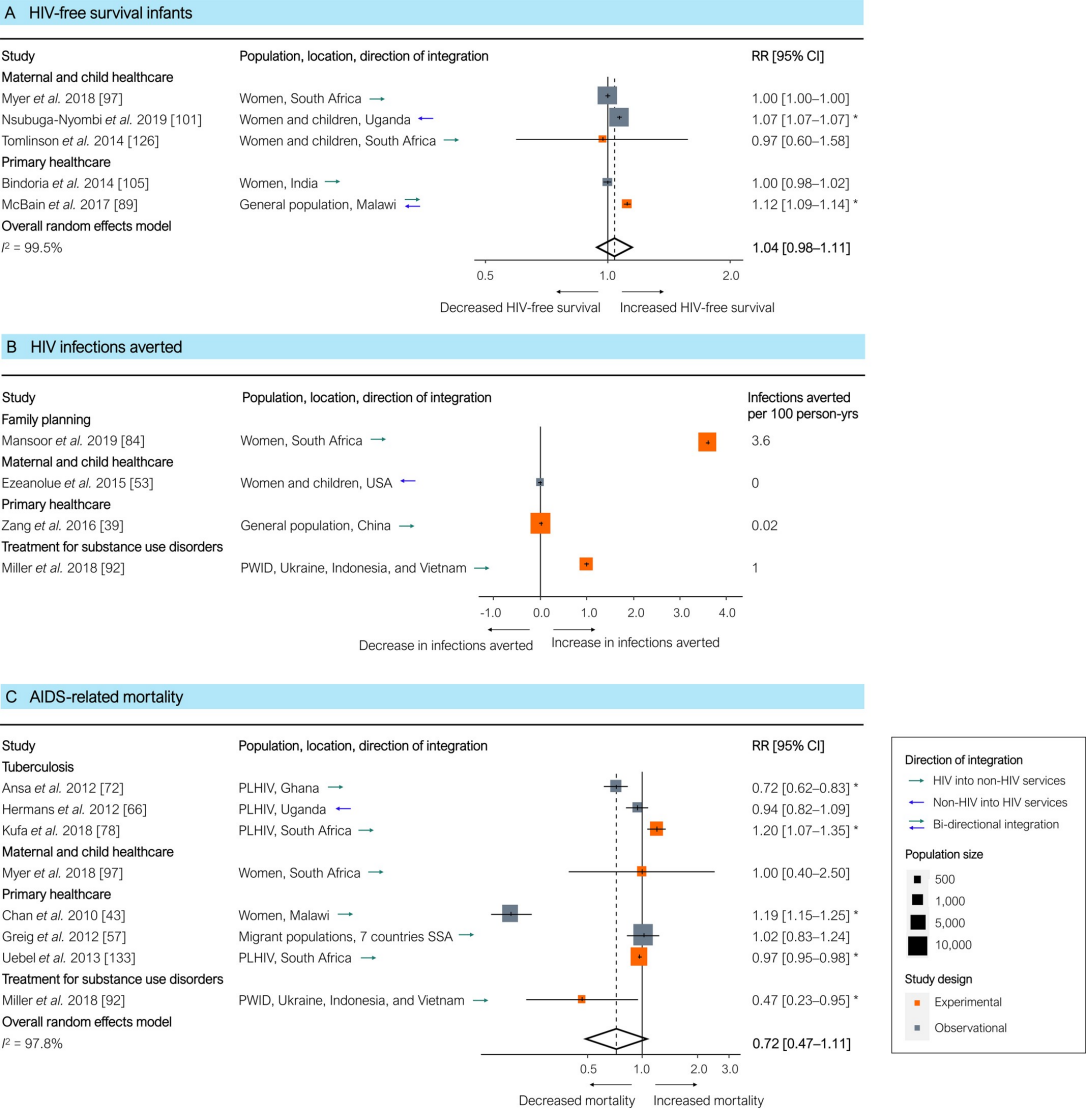
Results of HIV service integration: HIV-free survival among infants, HIV infections averted, and AIDS-related mortality. (A) HIV-free survival among infants. (B) HIV infections averted. (C) AIDS-related mortality. Outcomes are related to the ‘getting to zero’ targets for HIV/AIDS. Each estimate in (A) and (C) indicates the effect size as derived from a single study, either directly or by recalculating reported outcomes. Each estimate indicates the size of the relationship between integration exposure and outcome. We measured these relationships as RRs; asterisks indicate statistically significant results. The diamond at the bottom of each panel shows the overall random-effects meta-analytical estimate. Infections averted (B) are shown in 100 person-years. Abbreviations: CI, confidence interval; PLHIV, people living with HIV; PWID, people who inject drugs; RR, risk ratio; SSA, sub-Saharan Africa.

HIV-free survival among infants was only modestly and non-significantly higher in integrated programmes based on all 5 studies (pooled RR 1.04 [95% CI 0.98–1.11], *p =* 0.135; Fig 5A), but it was significantly higher based on the 2 experimental studies only (pooled RR 1.11 [95% CI 1.03–1.20], *p =* 0.033). Reported explanations for the moderate improvements in HIV-free survival outcomes were a low overall prevalence of mother-to-child transmission of HIV [97,105], national scale-up of ART for prevention of mother-to-child transmission [126], and other events promoting HIV testing and treatment initiation during the intervention period [105]. The number of HIV infections averted in integrated compared to separate services was similar in 2 studies and higher in 2 other studies (Fig 5B). AIDS-related mortality, measured in 8 studies, was non-significantly lower in integrated programmes (pooled RR 0.72 [95% CI 0.47–1.11], *p =* 0.118; Fig 5C). The increased mortality in integrated services in 1 study was attributed to weak implementation of integration and lack of coordination of integrated service delivery, affecting overall quality of care [78]. One study provided quantitative comparative outcomes related to the third UNAIDS ‘getting to zero’ target of ‘zero discrimination’ [3]. In this study, the integrated delivery of clinical HIV services and psychosocial interventions for adolescents in Zambia was significantly associated with reduced stigma (adjusted prevalence rate ratio 0.49 [95% CI 0.28–0.88]) and reduced negative community attitudes towards HIV (adjusted prevalence rate ratio 0.77 [95% CI 0.62–0.96]) [145].

### Non-HIV-related outcomes

**Fig 6:**
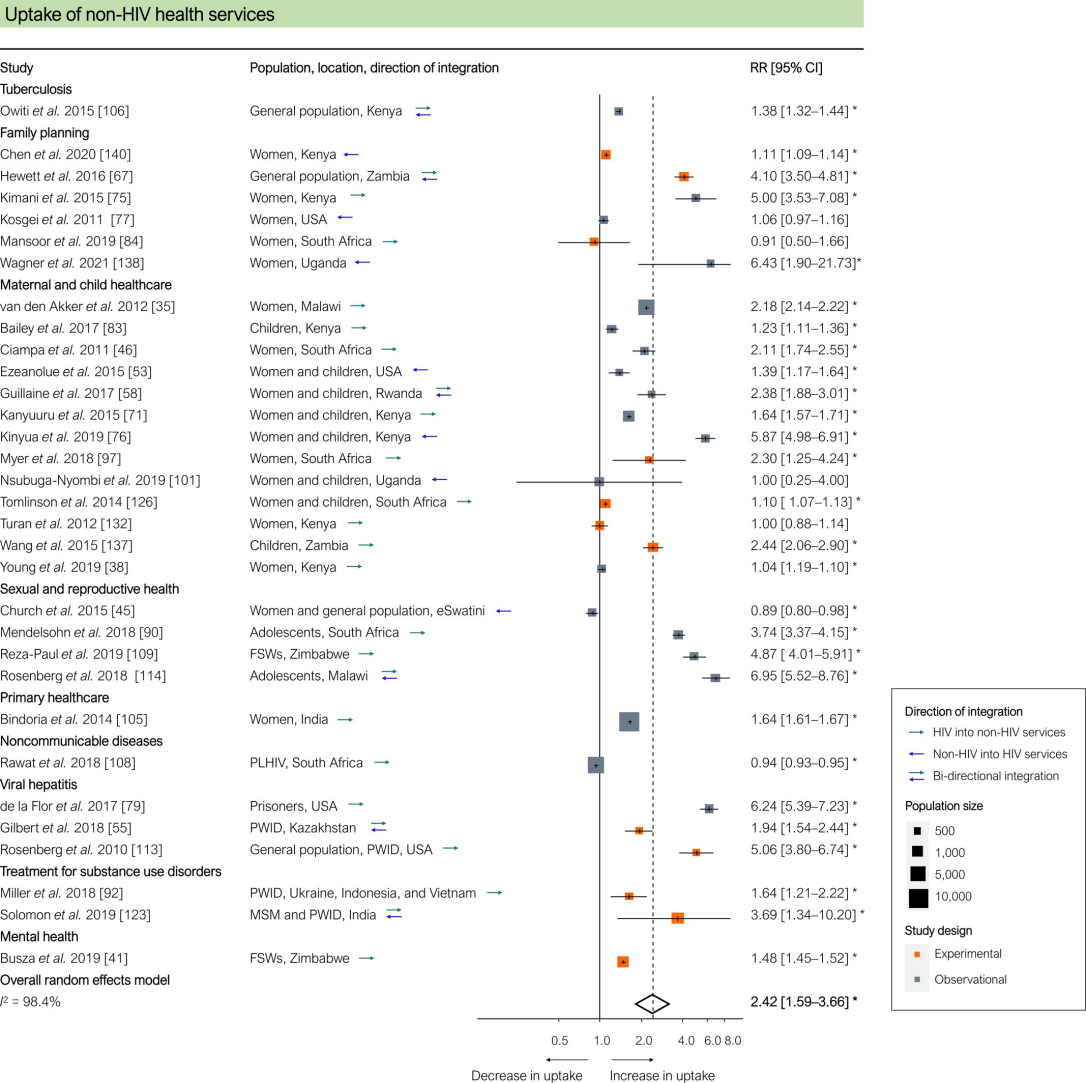
Results of HIV service integration: Uptake of non-HIV health services. Each estimate indicates the effect size as derived from a single study, either directly or through recalculating reported outcomes. Each estimate indicates the size of the relationship between integration exposure and outcome. We measured these relationships as RRs; asterisks indicate statistically significant results. The diamond at the bottom of each panel shows the overall random-effects meta-analytical estimate. Abbreviations: CI, confidence interval; FSWs, female sex workers; MSM, men who have sex with men; PLHIV, people living with HIV; PWID, people who inject drugs; RR, risk ratio.

**Fig 7:**
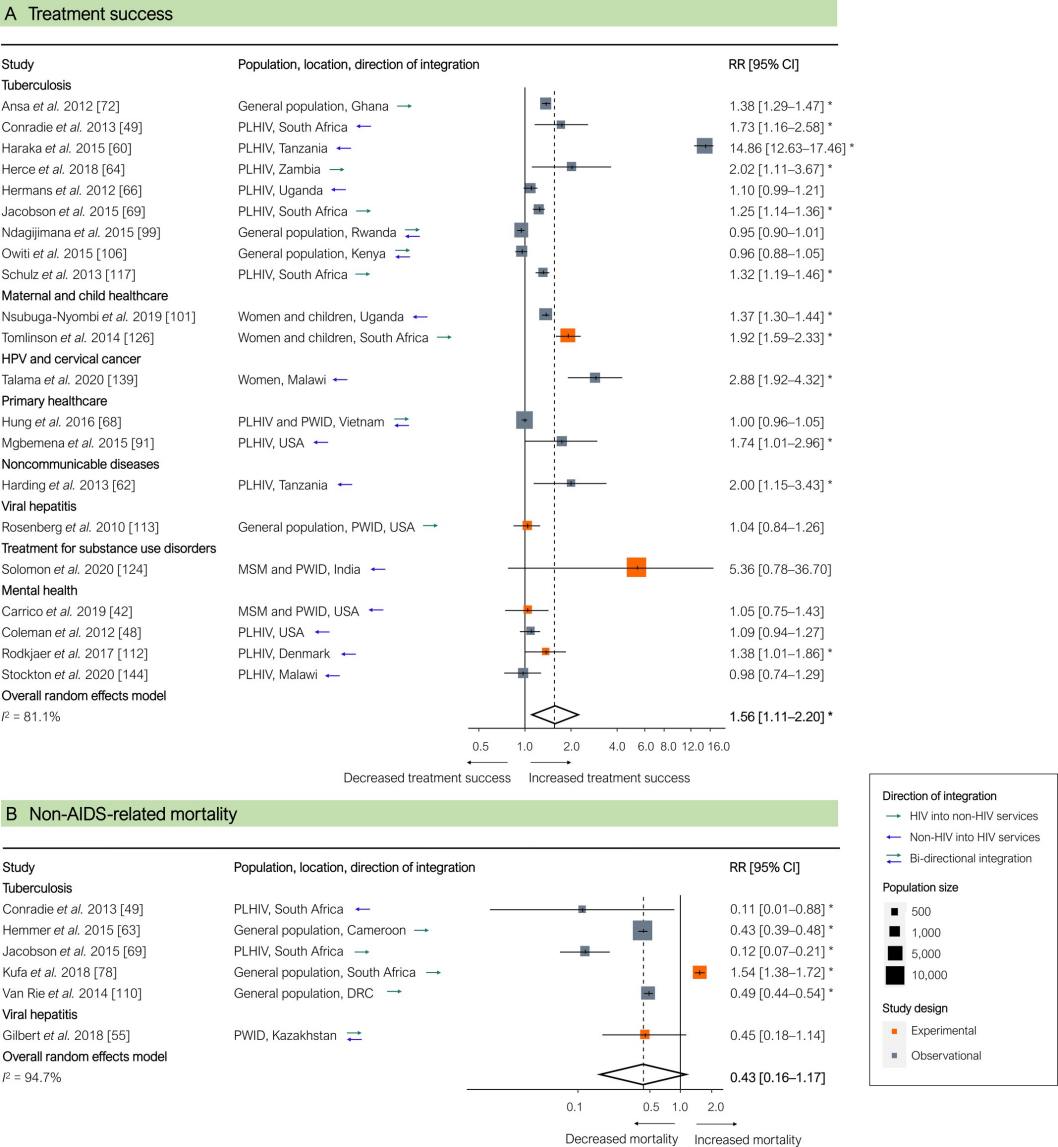
Results of HIV service integration: Treatment success for non-HIV-related diseases and conditions and non-AIDS-related mortality. (A) Treatment success for non-HIV related diseases and conditions. (B) Non-AIDS-related mortality. Each estimate indicates the size of the relationship between integration exposure and outcome. We measured these relationships as RRs; asterisks indicate statistically significant results. The diamond at the bottom of each panel shows the overall random-effects meta-analytical estimate. CI, confidence interval; DRC, Democratic Republic of the Congo; MSM, men who have sex with men; PLHIV, people living with HIV; PWID, people who inject drugs; HPV, human papillomavirus; RR, risk ratio; SSA, sub-Saharan Africa.

Uptake of other health services, measured in 32 studies, was significantly higher in integrated programmes (pooled RR 2.42 [95% CI 1.59–3.66], *p <* 0.001; Fig 6). In 2 studies, uptake of non-HIV services was significantly lower in integrated services, which the authors attributed to high HIV client loads, insufficient staff training to provide broader service packages, and insufficient human resource capacity to provide additional services [45,108]. Treatment success for diseases or conditions other than HIV, measured in 21 studies, was significantly higher in integrated programmes (pooled RR 1.56 [95% CI 1.11–2.20], *p =* 0.014; Fig 7A). Mortality from causes other than HIV (either TB or viral hepatitis), measured in 6 studies, was non-significantly lower in integrated programmes (pooled RR 0.43 [95% CI 0.16–1.17], *p =* 0.083; Fig 7B).

### Costs and cost-effectiveness

**Fig 8:**
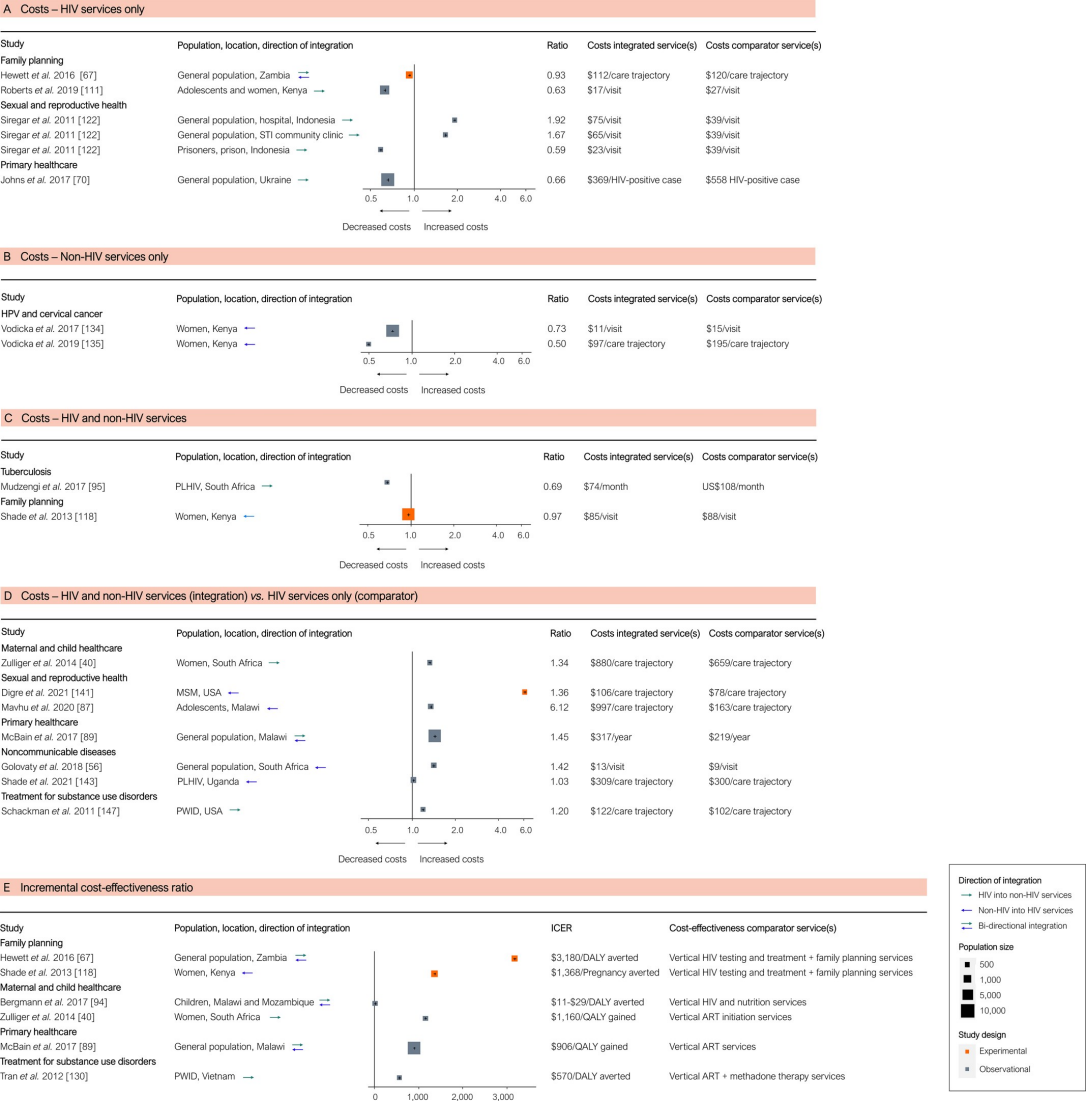
Results of integration of HIV services and economic outcomes: Costs and cost-effectiveness. (A–D) Costs of (A) HIV services only, (B) non-HIV services only, (C) HIV and non-HIV services combined, and (D) integrated non-HIV and HIV services compared to HIV services only. (E) Cost-effectiveness as ICERs. Each cost estimate indicates the effect size derived from a single study, either directly or through recalculating reported outcomes. The estimates represent the costs of services in integrated compared to separate services. The ICERs measure the cost-effectiveness of integration, compared to the cost-effectiveness of stand-alone HIV service delivery as reported in the studies. Abbreviations: ART, antiretroviral therapy; DALY, disability-adjusted life year; HPV, human papillomavirus; ICER, incremental cost-effectiveness ratio; MSM, men who have sex with men; PLHIV, people living with HIV; PWID, people who inject drugs; QALY, quality-adjusted life year; STI, sexually transmitted infection.

We found that costs of basic HIV and non-HIV services, measured in 10 studies, tended to be lower in integrated programmes (Fig 8). The ICERs of integrated services were universally positive, showing a wide range, from approximately US$10 to over US$3,000 per disability-adjusted life year averted or quality-adjusted life year gained (Fig 8E).

## Discussion

Our systematic review of integration of HIV services and other health services identified 114 relevant empirical peer-reviewed studies. Generally, outcomes were better in integrated compared to separate services: The meta-analytical averages for HIV testing and counselling, ART initiation, retention in HIV care, and viral suppression rates were all higher in integrated services. Similarly, uptake of non-HIV health services and non-HIV health outcomes were on average better in integrated services.

To our knowledge, this is the first systematic review and meta-analysis that quantifies the global evidence on integration of HIV services and other health services for a broad scope of health systems and health outcomes. Our systematic review was comprehensive in 4 important aspects. First, we included both directions of integration: HIV services integrated into services for other diseases or conditions, and services for diseases other than HIV—such as depression or hypertension screening and treatment—integrated into HIV services [17,26]. Second, we designed our search algorithm such that it identified all primary studies on the integration of HIV services with any other types of services. Third, we included a broad scope of outcomes in our review: HIV care cascade outcomes (testing, linkage to care, treatment initiation, treatment adherence, retention, and viral suppression), HIV health outcomes (new infections and mortality), non-HIV health outcomes, and costs and cost-effectiveness. Fourth, we included all study types as long as the study included a comparator of some sort for effect or impact estimation, i.e., we included experimental and observational quasi- and non-experimental studies in our review. Our results are broadly in line with earlier findings from systematic and scoping reviews on integration of services with narrower scopes of integration interventions or outcomes. Previous reviews on the integration of HIV and TB [148,149], MCH [22,150], family planning [151–153], SRH [154], primary healthcare [155], noncommunicable disease [23,151], and mental health [156] services generally found that integration led to improved service uptake and better health outcomes, but that high-quality evidence was limited [148,151–153,157–159]. Our synthesis also broadly concurs with the previous literature indicating that service integration evidence is particularly scarce for key populations [154], cost-effectiveness outcomes [156], and long-term impacts [19,20,151,156,160–162].

Our results should be useful for policy makers, practitioners, and researchers concerned with health system structures and processes supporting HIV services over the coming decades. Whether or not to integrate services and how to design integrated delivery models will likely depend on a wide range of factors, including the existing health system, resources, and HIV and other disease burdens. Although integration was generally successful, several specific integration interventions failed to improve outcomes. We identified several reasons why integration interventions were not successful. Integration failed to improve outcomes when it resulted in increased flows of more diverse patients in already overburdened health systems [45,108]. When integration remained limited to individual stages in the care cascade (for instance, when integration focused only on adding HIV testing or ART initiation) health outcomes did not improve in some studies, because of losses in other stages of the care cascade (such as retention and adherence) [34,82,98]. Other major reasons for integration failures were (i) imperfect fidelity of integration implementation (e.g., because of limited availability, training, or coordination of health workers) and (ii) low quality of care [45,56,78,82,108]. Overall, the studies identified in our review rarely reported on implementation fidelity, and if they did, fidelity was typically not quantified [45,78,82,98,108]. Future research should aim to capture both the fidelity of integration interventions, as well as meaningful and necessary local adaptations.

Quality of care is a critical element of studies of health service integration—it is plausible that integration either increases quality of care (because integration ensures that individual patients receive services that are well coordinated across multiple healthcare needs) or decreases quality of care (because integration decreases the specialization of service delivery structures and processes, leading to lower average quality of care for individual services). A key indicator of clinical quality of HIV care, viral suppression [163–165], consistently improved across different integration interventions covered in our systematic review, but with widely varying effect sizes. Data on other important aspects of quality of care were not available in the studies we identified in our systematic review. In particular, the studies covered did not provide estimates of the relationships between integration interventions and subjective perceptions of quality of care, the patient experience, and patient satisfaction. Such measures of peoples’ and patients’ subjective evaluations of health services are important—fundamentally, because they operationalize the health system outcomes of ‘patient satisfaction’ [166] and ‘responsiveness’ [167] and, instrumentally, because subjective evaluations influence health service uptake, retention, and adherence [168]. Future studies of integration of HIV services and other health services should include subjective measures and more detailed objective measures of quality of care. One promising approach is patient-reported outcome and experience measures (PROMs and PREMs) [169], and in particular PROM and PREM elicitation via digital devices.

Another important objective on the global HIV agenda is ‘zero discrimination’ [3]: We explicitly searched for outcomes of integration interventions related to discrimination and stigma, but found only 1 article that provided quantitative evidence [145]. However, emerging evidence suggests that interventions explicitly aimed at achieving ‘zero discrimination’ may produce greater population health benefits in jurisdictions with highly unequal access to care [170,171]. Such evidence will be important because integration could plausibly reduce discrimination by ‘normalising’ HIV services [172], but it may also cause discrimination, e.g., by reducing the privacy of vulnerable people [173,174]. Only 22 out of the 114 studies reported outcomes for key populations, demonstrating important knowledge gaps [159,175–181].

The current empirical evidence base on the impact of service integration on costs and cost-effectiveness does not provide insights into whether integration could indeed result in the hypothesized mitigation of the impact of declining resources for HIV. Our review showed that empirical evidence on the costs and cost-effectiveness of integration is limited and highly heterogeneous in terms of measured outcomes. For instance, some studies reported only HIV-related costs pre- and post-integration, while others reported only non-HIV costs. Furthermore, the complex trade-off between per-patient efficiency gains and overall programmatic costs requires careful attention when implementing integration strategies, yet these 2 components are rarely considered together. The existing empirical cost-effectiveness analyses [40,67,94] and modelling studies [182–191] show that, even if per-patient costs of service delivery go down, the increase in patient volume due to integration increases overall programme costs and resource needs. Finally, none of the studies assessed the overall healthcare quality impact of integration alongside changes in costs: Especially if resources are more stretched due to integration, per-patient costs may be lower, but so may be quality of care. Advancing our understanding of the economic impacts of integration will require more experimental or quasi-experimental studies that combine top-down macro-costing and bottom-up micro-costing approaches with quality and impact estimates of service delivery, e.g., through measuring health outcomes and conducting satisfaction surveys among patients and providers [192,193].

Our study had several limitations. First, as with any systematic review, the outcomes of our review are prone to publication bias, because studies reporting clearly positive findings may be more likely to be published than studies reporting null or negative findings. Second, the exclusion of case reports and ‘grey’ literature (e.g., project reports, conference abstracts) may have limited the range of evidence, especially for novel integration models. For example, our review excluded descriptions of HIV service integration into primary healthcare in Latin America [194], provision of hormone therapy alongside HIV services for transgender people [173], and HPV vaccination programmes integrated into HIV prevention and testing at secondary schools. Third, the experimental and observational primary studies that we synthesize in our meta-analysis may be biased. Findings from experimental studies provide the highest quality comparison, but may not be generalisable outside research settings. Evidence from quasi-experimental studies could remedy this situation by providing evidence with both high internal and high external validity [195,196]. Although some studies had relatively long study durations (4 years or more), none of them specifically reported on sustainment of the effects of service integration. Despite these limitations, for many outcomes our synthesis is encouraging, because the magnitudes of effects were large and consistent across the different study types.

In conclusion, our results support the integration of HIV services with other health services. The evidence indicates that integration of HIV testing and counselling services into non-HIV health programmes for people at risk of acquiring HIV, and integration of non-HIV services into ART programmes for PLHIV, tends to lead to improved service uptake and health benefits for HIV and other diseases or conditions. However, the effects of integrating HIV services into broader health systems and the economic impacts of integration are less clear and require further study. In addition, integration success will depend on a wide range of determinants and path dependencies, such as local epidemics and health system structures and processes. Despite the need for more studies on specific integration opportunities and key populations, it seems likely that integration of HIV and other health services can contribute to reaching the UNAIDS target of ending AIDS by 2030, while simultaneously meeting other universal healthcare targets.

## Supporting information

S1 PRISMA Checklist (PDF)

S1 Fig Flow chart of study selection. (PDF)

S2 Fig Study durations of the included primary studies. Figure shows the duration of studies on the *x*-axis (by bins, each indicating minimum up to and including the maximum duration) and the number of studies per bin on the *y*-axis.
(PDF)

S1 File Search strategy. (PDF)

S1 Table Characteristics of included primary studies. (PDF)

S2 Table Characteristics of studies that evaluated outcomes of integration of HIV services with other health services. Abbreviations: ANC, antenatal care; ART, antiretroviral therapy; CHWs, community health workers; EIMC, early infant male circumcision; HCV, hepatitis C virus; MCH, maternal and child health; MSM, men who have sex with men; NCDs, noncommunicable diseases; PMTCT, prevention of mother-to-child transmission; PNC, postnatal care; PWID, people who inject drugs; PrEP, pre-exposure prophylaxis; SRH, sexual and reproductive health; STI, sexually transmitted infection; TB, tuberculosis; VMMC, voluntary medical male circumcision.
(PDF)

S3 Table GRADE assessment of the quality of the included studies. (PDF)

## Abbreviations

ART: antiretroviral therapy
GRADE: Grading of Recommendations Assessment, Development and Evaluation
ICER: incremental cost-effectiveness ratio
MCH: maternal and child health
PLHIV: people living with HIV
RR: risk ratio
SRH: sexual and reproductive health
STI: sexually transmitted infection
TB: tuberculosis
