# Supplementary figures and images for: Integrating HIV services and other health services: A systematic review and meta-analysis
Source: PLoS Med. 2021 Nov 9;18(11):e1003836. doi: 10.1371/journal.pmed.1003836 (PMC8577772; doi:10.1371/journal.pmed.1003836)

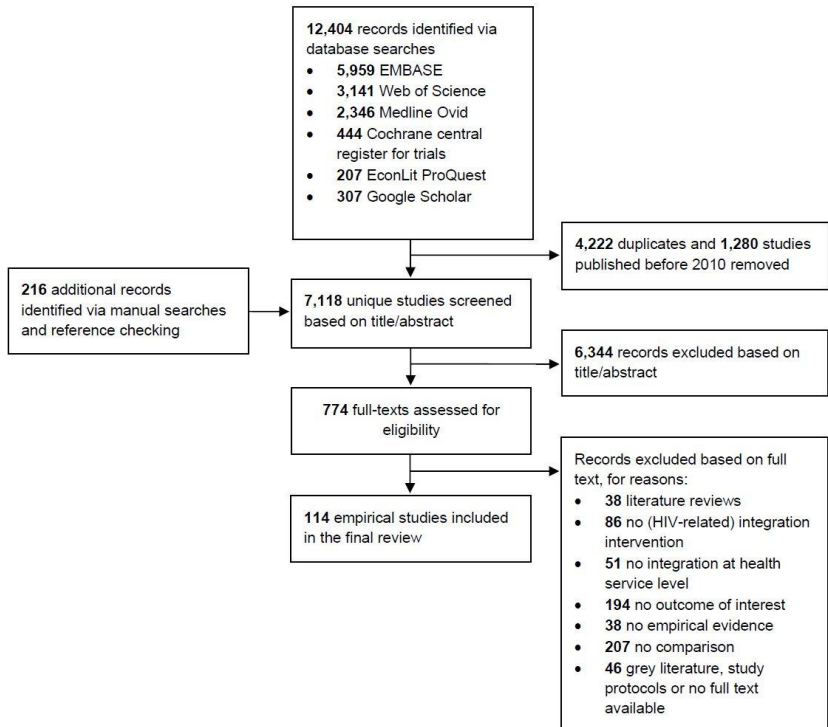

Supplement: S1 Fig — (PDF) [file pmed.1003836.s002.pdf]

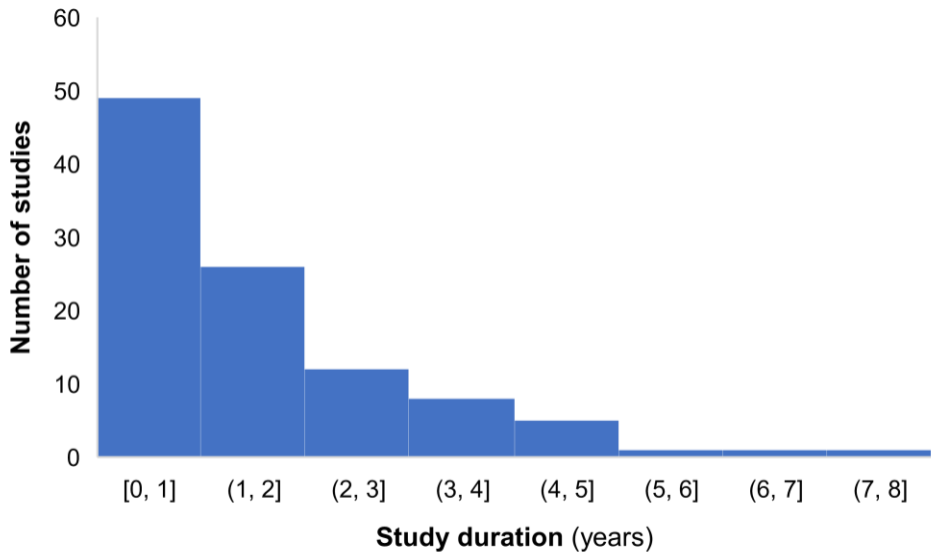

Supplement: S2 Fig — Figure shows the duration of studies on the x-axis (by bins, each indicating minimum up to and including the maximum duration) and the number of studies per bin on the y-axis. (PDF) [file pmed.1003836.s003.pdf]
