# Supplementary material for: Integrating HIV services and other health services: A systematic review and meta-analysis
Source: PLoS Med. 2021 Nov 9;18(11):e1003836. doi: 10.1371/journal.pmed.1003836 (PMC8577772; doi:10.1371/journal.pmed.1003836)
Supplement: S1 File — (PDF) [file pmed.1003836.s004.pdf]

S1 File. Search strategy.

Searches performed 28 January 2020, updated searches performed 10 September 2021; search terms were adapted to fit requirements by each database.

1. ('integrated health care system'/de OR ((integrat* OR coordinat* OR interdisciplin* OR inter-disciplin*) NEAR/8 (service* OR care OR healthcare OR deliver* OR hospital* OR public-health OR program* OR health-system* OR routine* OR intervention* OR centre* OR center*)):ab,ti OR (deliver* NEAR/3 model*):ti)

2. AND ('Human immunodeficiency virus infection'/exp OR 'Human immunodeficiency virus'/exp OR 'acquired immune deficiency syndrome'/exp OR 'highly active antiretroviral therapy'/exp OR (hiv OR aids OR haart OR (Human NEAR/3 (immunodeficien* OR immuno-deficien* OR immune-deficien*) NEAR/3 virus*) OR (acquir* NEAR/3 (immunodeficien* OR immuno-deficien* OR immune-deficien*) NEAR/3 syndrome*) OR antiretroviral-therap* OR anti-retroviral-therap*):ab,ti)

3. AND ('economic aspect'/exp OR economics/de OR 'health economics'/exp OR 'cost'/de OR 'health care cost'/exp OR 'disability-adjusted life year'/de OR 'quality adjusted life year'/de OR 'health care utilization'/exp OR 'facilities and services utilization'/de OR 'health care quality'/de OR 'clinical effectiveness'/de OR 'health equity'/de OR 'clinical indicator'/de OR benchmarking/de OR 'personnel management'/de OR 'quality improvement study'/de OR 'total quality management'/de OR 'feasibility study'/de OR (economic* OR cost OR costs OR financ* OR ((disabilit* OR qualit*) NEAR/3 adjust* NEAR/3 (life-year* OR lifeyear*)) OR daly* OR qaly* OR ((service* OR healthcare OR care OR equipment* OR supplies* OR procedure* OR technique*) NEAR/3 (utili* OR uptake*)) OR ((clinical* OR care OR healthcare OR service*) NEAR/3 (effectiv* OR efficien* OR indicator*)) OR ((qualit* OR performance*) NEAR/3 (indicator* OR measure* OR improve* OR management*)) OR equity OR benchmark* OR ((personnel OR staff) NEAR/3 (management* OR utili*)) OR utilization* OR utilization* OR feasib* OR sustainab*):Ab,ti OR quality:ti) NOT ([Conference Abstract]/lim)

4. AND ('Human immunodeficiency virus infection'/exp OR 'Human immunodeficiency virus'/exp OR 'acquired immune deficiency syndrome'/exp OR 'highly active antiretroviral therapy'/exp OR 'transgender'/exp OR 'sex worker'/exp OR 'prostitution'/exp OR 'men who have sex with men'/de OR 'sexual and gender minority'/exp OR (hiv OR aids OR haart OR (Human NEAR/3 (immunodeficien* OR immuno-deficien* OR immune-deficien*) NEAR/3 virus*) OR (acquir* NEAR/3 (immunodeficien* OR immuno-deficien* OR immune-deficien*) NEAR/3 syndrome*) OR antiretroviral-therap* OR anti-retroviral-therap* OR transgender* OR trans-gender* OR trans-people* OR sex-work* OR sex-traffick* OR prostitut* OR ((men-who OR men-having OR men-reporting) NEAR/3 sex-with-men) OR lgbtq):ab,ti)

5. AND [English]/
