# Supplementary material for: Integrating HIV services and other health services: A systematic review and meta-analysis
Source: PLoS Med. 2021 Nov 9;18(11):e1003836. doi: 10.1371/journal.pmed.1003836 (PMC8577772; doi:10.1371/journal.pmed.1003836)
Supplement: S1 Table — (PDF) [file pmed.1003836.s005.pdf]

S1 Table. Characteristics of included primary studies.

	Total number of studies[1]	HIV testing and/or counselling (and/or secondary prevention)	Antiretroviral treatment (ART)
**All included studies**	**114**	**60**	**54**
**Study design**			
Experimental studies: randomised controlled trials	13	6	7
Experimental studies: cluster-randomised controlled trials	17	10	7
Experimental studies: non-randomised trials	9	7	2
Quasi-experimental studies: pre-post intervention studies, time series analyses	26	16	11
Observational comparison studies: prospective or retrospective cohort studies	36	15	21
Observational comparison studies: cross-sectional studies	11	6	6
**Geographical region**			
Eastern and Southern Africa	79	42	7
Western and Central Europe and North America	13	7	7
Southeast Asia and the Pacific	11	6	6
West and Central Africa	5	3	2
Eastern Europe and Central Asia	4	2	2
**Health service area**			
Maternal and child healthcare	28	16	13
Tuberculosis	16	6	10
Primary healthcare	14	5	9
Family planning	16	14	2
Sexual and reproductive health and STIs	13	10	3
Substance use therapy	9	4	6
Non-communicable diseases	5	2	3
Mental health	5	0	5
Viral hepatitis	3	2	1
HPV/cervical cancer	3	1	2
**Target population**			
General population or PLHIV within the general population (*e.g.,* HIV/TB co-infected people)	50	26	24
Women and/or children	40	22	19
Adolescents	6	4	2
Men who have sex with men (MSM)	4	3	1
Transgender people	0	0	0
Sex workers	2	0	2
People who inject drugs (PWID)	8	4	5

Prisoners and incarcerated people	1	1	0
Migrants and displaced people	1	0	1
**Direction of integration**			
HIV services into non-HIV programmes (including broader primary healthcare programmes)	67	46	21
Non-HIV services into HIV programmes	36	9	29
Bi-directional integration (or newly set-up services)	9	5	4
**Healthcare setting**			
Hospital(s), outpatient clinic(s), emergency clinic(s)	22	12	10
Local public health clinic(s)	51	22	30
Community-level	20	10	11
Multiple settings, other type(s) of setting(s)	19	16	3
**Urban/rural**			
Urban	38	21	18
Semi-urban	7	3	4
Rural	25	13	13
Mixed	29	20	9
Unknown	13	3	10

[1]Primary subgroups shown, whereas studies could fall into multiple subgroups.
