# Supplementary material for: Integrating HIV services and other health services: A systematic review and meta-analysis
Source: PLoS Med. 2021 Nov 9;18(11):e1003836. doi: 10.1371/journal.pmed.1003836 (PMC8577772; doi:10.1371/journal.pmed.1003836)
Supplement: S2 Table — Abbreviations: ANC, antenatal care; ART, antiretroviral therapy; CHWs, community health workers; EIMC, early infant male circumcision; HCV, hepatitis C virus; MCH, maternal and child health; MSM, men who have sex with men; NCDs, noncommunicable diseases; PMTCT, prevention of mother-to-child transmission; PNC, postnatal care; PWID, people who inject drugs; PrEP, pre-exposure prophylaxis; SRH, sexual and reproductive health; STI, sexually transmitted infection; TB, tuberculosis; VMMC, voluntary medical male circumcision. (PDF) [file pmed.1003836.s006.pdf]

S2 Table. Characteristics of studies that evaluated outcomes of HIV services integration with other health services. Abbreviations: ANC = antenatal care; ART = antiretroviral therapy; CD4 = cluster of differentiation 4; CHWs = community health workers; EIMC = early infant male circumcision; HCV = hepatitis C virus; HIV = human immunodeficiency virus; MCH = maternal and child healthcare; MSM = men who have sex with men; NCDs = non-communicable diseases; PMTCT = prevention-of-mother-to-child-transmission; PNC = postnatal care; PWID = people who inject drugs; PrEP = pre-exposure prophylaxis; SRH = sexual and reproductive health; STIs = sexually transmitted infections; TB = tuberculosis; VMMC = voluntary male medical circumcision.

Author(s) and year of publication	HIV services integration area	Target population	Location	Study period	Study design	Intervention(s), comparison(s)	Healthcare setting	Sample size	Outcome measures
Akinleye *et al.* 2017 [34]	Maternal and child healthcare	Pregnant women and children below five years	Nigeria (Benue State)	2014	Cross-sectional study	Integration of HIV testing and PMTCT services into MCH weeks (mainly ANC provision)	Community-level (rural)	50,253 (at multiple sites)	(1) New HIV cases identified (2) Linkage to PMTCT (3) Service uptake
Akker *et al.* 2012 [35]	Maternal and child healthcare, family planning, SRH and STIs	Women attending reproductive health services	Malawi (Thyolo)	2005-10	Pre-post study	Integration of PMTCT into MCH, family planning and SRH services	Local public health clinic(s) (unknown)	53,330 (at multiple sites)	(1) Uptake of SRH, antenatal services (2) HIV incidence among infants (3) Utilisation of PMTCT services
Aliyu *et al.* 2016 [50]	Maternal and child healthcare	HIV-positive pregnant women presenting for antenatal care	Nigeria (Niger State)	2013-14	Randomized controlled trial	Integration of HIV testing and counselling, CD4 testing, PMTCT, infant feeding, home-based primary care and early infant HIV testing at primary and secondary level healthcare facilities	Local public health clinic(s) (rural)	369 (at 12 sites)	(1) ART adherence (2) Retention in care
Ameh *et al.* 2017 [61]	NCDs	PLHIV accessing HIV care	South Africa (Bushbuckridge)	2011-13	Pre-post study (controlled interrupted time-series)	Integration of chronic disease management into ART programmes at primary healthcare clinics	Local public health clinic(s) (rural)	870 (at 21 sites)	(1) Control of CD4 counts (2) Control of hypertension
Ansa *et al.* 2012 [72]	Tuberculosis	TB-positive people already accessing TB services	Ghana (Eastern region)	2006-08	Pre-post study	Integration of collaborative HIV/TB activities into existing TB services through one-stop-shop, partially integrated site or referral site	Hospital(s) (urban, rural)	1,330 (at 3 sites)	(1) TB health outcomes (2) Retention in care
Bailey *et al.* 2017 [83]	Maternal and child healthcare	Parents and male infants	Kenya (Homa Bay County)	2014-16	Non-randomised trial	Integration of HIV screening and community-level early infant male circumcision (EIMC) promotion and provision into MCH services	Community-level (urban, rural)	2,117 (at 16 sites)	(1) EIMC acceptability (2) EIMC uptake

Bergmann *et al.* 2017 [94]	Maternal and child healthcare; nutrition	HIV-exposed infants under five years of age	Malawi, Mozambique	2011-13	Pre-post intervention study	Integration of HIV testing, PMTCT and nutrition service delivery into one-stop-shops with additional innovative healthcare components (*e.g.*, male motivators, SMS services)	Multiple settings (unknown)	N/A (at multiple sites)	(1) Numbers of lives saved,<br>(2) Infections averted<br>(3) Undernutrition cases cured<br>(4) Cost-effectiveness<br>(5) Enrolment in ART programmes
Bindoria et a*l.* 2014 [105]	Primary healthcare	Pregnant women	India (Maharashtra)	2010-12	Pre-post study	Integration of HIV screening into primary healthcare clinics providing antenatal services and linkage to ART delivery programmes	Local public health clinic(s) (rural)	105,650 (at 36 sites)	(1) Feasibility<br>(2) Relative benefits<br>(3) Incremental cost-effectiveness ratio
Broughton *et al.* 2016 [116]	Primary healthcare; non-communicable diseases	PLHIV	Uganda (Mityana and Nakaseke)	2013-14	Pre-post intervention study (difference-in-difference)	Integration of a chronic disease care model into ART programmes	Local public health clinic(s) (rural)	102 (at 3 sites	(1) ART adherence<br>(2) CD4 counts<br>(3) Incremental cost-effectiveness ratio
Brunie *et al.* 2016 [127]	Family planning	General population accessing family planning services	Uganda	2012-13	Cluster-randomized controlled study	Integration of HIV testing and counselling into existing family planning services	Community-level (rural)	137 (at 1 site)	(1) Feasibility<br>(2) Acceptability<br>(3) Quality of care
Brunie *et al.* 2017 [36]	Family planning	General population accessing family planning services	Uganda	2012-13	Cluster-randomized controlled study	Integration of HIV testing and counselling into existing family planning services	Community-level (rural)	256 (at 1 site)	(1) Feasibility<br>(2) Acceptability<br>(3) Uptake of HIV and FP services<br>(4) Time spent on healthcare provision
Busza *et al.* 2019 [41]	Mental health; SRH and STIs; PrEP	Female sex workers	Zimbabwe	2014-16	Randomized controlled trial	Integration of HIV testing, ART, adherence counselling, PrEP, psychosocial support, into existing SRH services (SAPPH-IRe trial, Sisters with a Voice)	Community-level (semi-urban)	8,231 (at 28 sites)	(1) HIV-tests performed<br>(2) Number of new HIV-cases diagnosed<br>(3) Programme implementation fidelity
Carrico *et al.* 2019 [42]	Mental health	PLHIV who fall under MSM and/or PWID	USA (San Francisco)	2013-17	Randomized controlled trial	Integration of positive affect intervention or attention-control programmes into ART adherence programmes, financial incentives for abstinence	Community-level (urban)	110 (at 1 site)	(1) HIV viral load at 6, 12 and 15 months<br>(2) Risk of unsuppressed HIV RNA (>200 copies/mL) over 15 months<br>(3) Positive affect, self-reported frequency of stimulant use at 6 and 12 months

Chan *et al.* 2010 [43]	Primary healthcare	PLHIV registered for ART services	Malawi (Zomba District)	2008	Retrospective cohort study	Integration of ART services into broader basic primary care clinics – decentralised model	Local public health clinic(s) (rural)	8,093 (at 24 sites)	(1) (Equity in) access to HIV services<br>(2) ART adherence<br>(3) AIDS-related mortality
Chan *et al.* 2016 [44]	Maternal and child healthcare; PMTCT	Pregnant women seeking ANC at primary health facilities	Malawi (Zomba District)	2011-12	Cohort study	Integration of multi-disease testing, ANC into HIV pogrammes (HIV testing and counselling, ART provision)	Local public health clinic(s) (rural)	10,528 (at 23 sites)	(1) ART uptake rates<br>(2) Retention rates
Chen *et al.* 2020 [140]	Family planning	Women visiting routine HIV care and treatment	Kenya	2016	Cross-sectional study	Integration of family planning services into HIV clinics	Local public health clinic(s) (mixed)	(at 108 sites)	(1) Volume of integrated family planning services into HIV services<br>(2) Uptake of contraceptives
Church *et al.* 2015 [45]	SRH and STIs; family planning	Female (of reproductive age) and male (all ages) HIV service clients	eSwatini (Manzini)	2009	Non-randomised trial	Integration of different SRH and family planning delivery models into HIV care	Local public health clinic(s) (semi-urban)	602 (at 4 clinics)	(1) Unmet family planning service needs
Ciampa *et al.* 2011 [46]	Maternal and child healthcare	HIV-positive pregnant women and/or women with young infants presenting at MCH services	Mozambique (Zambezia Province)	2009-10	Cohort Study	Integration of HIV testing of exposed infants into MCH locations	Hospital(s) (rural)	395 (at 2 sites)	(1) Retention in care of infants<br>(2) Infant testing timing
Click *et al.* 2012 [47]	Tuberculosis	Children registered at TB clinics	Ethiopia	2007-09	Pre-post study	Integration of HIV testing into TB services	Multiple settings (mixed)	496 (at 8 sites)	(1) Documented HIV rapid test results among children
Coleman *et al.* 2012 [48]	Mental health	PLHIV: HIV patients suffering from depression	USA (Boston)	2004-10	Pre-post intervention study	Integration of depression care into ART programmes	Hospital(s) (unknown)	124 (at 1 site)	(1) Depression score<br>(2) HIV viral loads and CD4 counts<br>(3) Antidepressant prescription
Conradie *et al.* 2013 [49]	Tuberculosis	HIV/TB co-infected patients presenting at ART clinics	South Africa (Breede Valley)	2009-10	Retrospective cohort study	Integration of TB care into ART services at infectious disease clinic	Local public health clinic(s) (unknown)	100 (at 1 site)	(1) TB treatment completion<br>(2) TB treatment success<br>(3) TB mortality
Criniti *et al.* 2011 [51]	Family planning	Women visiting urban family planning and adult HIV care clinic in the same university system	USA (Philadelphia, Pennsylvania)	2007-09	Cohort study	Integration of routine HIV testing and counselling into family planning services with gynaecologic and prenatal care services and psychosocial support	Hospital(s) (urban)	2,185 (at 1 site)	(1) Percentage of patients with HIV test in the last 12 months<br>(2) Testing acceptance rate<br>(3) New HIV cases identified

									(4) Feasibility and Acceptability
Deo *et al.* 2012 [52]	Primary healthcare	Outpatient department patients	Zambia (Lusaka)	2008-09	Prospective cohort study	Integration of HIV testing and treatment services into outpatient care at a primary care facility	Outpatient-clinic(s) (urban)	80 (at 1 site)	(1) Patient waiting times
De la Flor *et al.* 2017 [79]	Viral hepatitis	Prisoners and incarcerated people: inmates entering jail	USA (Dallas, Texas)	2015-16	Pre-post study	Opt-out testing for HIV into routine HCV testing during intake	Other (urban)	3,155 (at 1 site)	(1) Service uptake<br>(2) New HIV/HCV cases identified
Digre *et al.* 2021 [141]	SRH and STIs; PrEP	STI clinics	USA (Mississippi)	2016-18	Pre-post study	Integration of HIV testing and PrEP for partners at STI clinics	Local public health clinic(s) (urban)	(at 3 sites)	(1) Uptake of HIV services<br>(2) Marginal costs per case
Dovel *et al.* 2020 [142]	Outpatient services	Outpatient clinics	Malawi (Central and Southern areas)	2017-18	Cluster-randomized controlled trial	Integration of HIV self-testing into outpatient clinics	Local public health clinic(s) and hospitals (mixed)	5,885 (at 15 sites)	(1) Uptake HIV testing<br>(2) Costs
Ezeanolue *et al.* 2015 [53]	Maternal and child healthcare; PMTCT	Women and infants who are HIV-positive or HIV-exposed	USA (Las Vegas, Nevada)	2007-12	Retrospective cohort study	Integration of prenatal care and in-facility delivery services into HIV testing and treatment services	Local public health clinic(s) (urban)	131 (at 1 site)	(1) Adequacy of prenatal care<br>(2) Appropriate ART use<br>(3) caesarean section uptake<br>(4) Mother-to-child HIV transmission rate
Geelhoed *et al.* 2013 [54]	Maternal and child healthcare; PMTCT	HIV-positive women and their HIV-exposed infants	Mozambique (Tete Province)	2009-10	Cluster-randomized controlled trial	Reorganising HIV testing and counselling and ART provision and MCH services into one-stop shops	Local public health clinic(s) (rural)	2,559 (at 6 sites)	(1) Feasibility<br>(2) Utilization rates<br>(3) Retention rates<br>(4) Service Quality
Gilbert *et al.* 2018 [55]	Viral hepatitis; treatment for substance use disorders	HIV/HCV co-infected PWID: people using heroin or other opioids and their HIV/HCV-negative injection partners	Kazakhstan (Almaty)	2009-13	Randomized controlled trial	Five-session couple-based HIV/HCV and overdose prevention intervention	Community-level (urban)	479 (at 1 site)	(1) Non-fatal overdose rates, heroin/opioid injection use<br>(2) Drug treatment attendance rates, naloxone use<br>(3) Overdose death rates
Golovaty *et al.* 2018 [56]	Non-communicable diseases	General population: adult inhabitants of rural community	South Africa (KwaZulu-Natal)	2012-15	Cross-sectional study nested in prospective cohort study	Integration of NCD screening and linkage to HIV care into home-based HIV testing and counselling	Community-level (rural)	570 (in one community)	(1) Incremental costs<br>(2) Efficiency of utilized resources

Greig *et al.* 2012 [57]	Primary healthcare; tuberculosis; SRH and STIs	HIV-positive migrant and refugee patients on ART	Sub-Saharan Africa (9 countries)	2003-10	Retrospective cohort study	Integration of ART programmes into general healthcare clinics (through Medicines Sans Frontiers services)	Community-level (rural)	15,403 (at 17 sites)	(1) AIDS-related mortality<br>(2) Risk of loss to follow-up
Guillaine *et al.* 2017 [58]	Maternal and child healthcare	HIV-positive mothers and their infants	Rwanda (Southern Kayonza and Kirehe Districts)	2012-13	Retrospective cohort study	Integration of maternal and child health services for HIV-positive women and their children – bi-directional	Local public health clinic(s) (rural)	185 (at 2 clinics)	(1) Service utilization<br>(2) Retention rates<br>(3) Linkage to routine services<br>(4) Clinical outcomes
Hankin *et al.* 2016 [59]	Primary healthcare	General population	USA	2008-13	Retrospective cohort study	Integration of non-targeted routine HIV screening into emergency triage process	Emergency clinic(s) (urban)	~120,000 (at 1 site)	(1) Number of HIV tests<br>(2) New diagnoses<br>(3) Feasibility
Haraka *et al.* 2015 [60]	Tuberculosis	HIV-positive patients without prior history of TB	Tanzania (Ifakara, Morogoro)	2005-13	Pre-post study	Integration of one-stop-shop TB testing and treatment into ART programmes (Kilombero Ulanga Antiretroviral Cohort, KIULARCO)	Community-level (rural)	5,123	(1) Incident TB cases among HIV patients<br>(2) TB ascertainment
Harding *et al.* 2012 [62]	Primary healthcare; non-communicable diseases	PLHIV presenting with pain symptoms	Tanzania (North East region)	2009-10	Prospective longitudinal controlled-intervention study	Integration of HIV outpatient palliative care into ART provision services	Outpatient-clinic(s) (unknown)	128 (at 1 site)	(1) Odds of reporting pain<br>(2) Treatment success
Hemmer *et al.* 2015 [63]	Tuberculosis	TB and HIV patients	Cameroon (Littoral Province)	2007-09	Pre-post study	Integration of HIV services into national TB programmes	Local public health clinic(s) (urban, rural)	~16,000 (at 30 sites)	(1) HIV testing rates<br>(2) Treatment prescribing rate for TB/HIV-patients<br>(3) Mortality of newly recruited TB patients
Herce *et al.* 2018 [64]	Tuberculosis	TB-positive PLHIV	Zambia (Lusaka)	2011-12	Pre-post study	Integration of HIV testing and treatment into one-stop-shop TB treatment and care	Local public health clinic(s) (urban)	473 (at 2 sites)	(1) Linkage to HIV care<br>(2) Early ART uptake<br>(3) TB treatment success
Herlihy *et al.* 2015 [65]	Maternal and child healthcare	HIV-positive pregnant women visiting governmental ANC clinics	Zambia (Southern Province)	2011-13	Cohort study	Integration of PMTCT into MCH services with community-based follow-up	Local public health clinic(s) (rural)	1,134 (at 1 site)	(1) CD4-testing rates<br>(2) Proportion ART-eligible<br>(3) HIV-exposed infant testing rates at 6 months
Hermans *et al.* 2012 [66]	Tuberculosis	PLHIV presenting at large HIV clinic	Uganda (Kampala)	2007-09	Pre-post study	Integration of TB testing and treatment services into one-stop-shop ART programmes and outpatient care	Local public health clinic(s) (urban)	712 (at 1 site)	(1) ART initiation<br>(2) CD4 counts<br>(3) TB treatment success
Hewett et al. 2016 [67]	Family planning; SRH and STIs	Adults accessing family planning,	Zambia (Lusaka and Chipata)	2013-15	Randomised trial	Integration of HIV testing and counselling with SRH services, family planning, VMMC, cervical cancer	Local public health clinic(s) (urban)	3,963 (at 3 sites)	(1) Uptake of services<br>(2) Cost-effectiveness

		HIV testing and/or VMMC services				screening – bi-directional models with referral to full integration			
Hung *et al.* 2016 [68]	Primary healthcare; treatment for substance use disorders	HIV-positive PWID and HIV-negative PWID	Vietnam (Ho Chi Minh City)	2013-14	Pre-post study	Integration of co-located voluntary HIV counselling and testing, treatment and methadone maintenance therapy using different integration models	Outpatient-clinic(s) (urban)	8,228 (at 7 sites)	(1) Linkage<br>(2) ART initiation<br>(3) Adherence
Jacobson *et al.* 2015 [69]	Tuberculosis	HIV/TB co-infected patients initiating treatment for TB	South Africa (KwaZulu-Natal)	2012-13	Retrospective cohort study	Integration of one-stop-shop HIV and TB treatment and care into primary health clinics or referral from HIV clinics – decentralised approach	Local public health clinic(s) (rural)	657	(1) TB treatment success<br>(2) Retention in care
Johns *et al.* 2017 [70]	Primary healthcare	Population of Chernigiv, Northern Ukraine	Ukraine (Chernigiv Province)	2013-15	Pre-post study (difference-in-difference)	Integration of HIV testing and counselling into primary healthcare	Outpatient-clinic(s) (urban, rural)	86,000 (at 20 sites)	(1) Incremental costs<br>(2) Number of newly detected HIV cases
Kanyuuru *et al.* 2015 [71]	Maternal and child healthcare	Pregnant women and their infants	Kenya (Bondo District)	2010-12	Pre-post intervention study	Integration of MCH, HIV and immunisation services into routine community programmes	Local public health clinic(s) (unknown)	People from 329 villages	(1) Uptake of prenatal services<br>(2) Uptake of HIV testing<br>(3) Access to care
Katz *et al.* 2016 [73]	SRH and STIs	Men who have sex with men presenting with STIs	USA (Washington)	2010-14	Cohort study	Integration of HIV testing and counselling for MSM with early syphilis, gonorrhoea or chlamydial infection and partner services into STI services in state hospitals	Outpatient-clinic(s) (urban)	8,133 (at multiple sites)	(1) HIV-testing rates for patients, partner services<br>(2) New HIV cases identified<br>(3) Proportion of new cases with concurrent STI diagnosis
Kerschberger *et al.* 2012 [74]	Tuberculosis	HIV/TB co-infected patients registered for TB treatment	South Africa (Cape Town)	2008-09	Pre-post study	Integration of one-stop-shop HIV and TB treatment and care into primary healthcare clinic	Local public health clinic(s) (semi-urban)	188 (at 1 site)	(1) ART uptake<br>(2) Time to ART treatment initiation
Kimani *et al.* 2015 [75]	Family planning	Women receiving postnatal care	Kenya (Eastern Kenya)	2010	Non-randomized trial	Integration of HIV and family planning services into postnatal care at MCH clinics (Integra Initiative)	Local public health clinic(s) (semi-urban)	1,204	(1) Health services uptake<br>(2) Effectiveness of integration
Kinyua *et al.* 2019 [76]	Maternal and child healthcare	HIV-positive mothers and their HIV-exposed infants	Kenya (Kwale County)	2013-16	Pre-post intervention study	Integration of nutrition assessment, counselling and support into PMTCT services at HIV clinics through Ministry of Health programme	Local public health clinic(s) (rural)	837 (at 16 sites)	(1) Uptake of nutrition counselling and support<br>(2) Retention of mother-baby-pairs

Kosgei *et al.* 2011 [77]	Family planning	HIV-positive women	Kenya (Western Kenya)	2005-09	Retrospective cohort study	Integration of family planning into routine HIV services	Local public health clinic(s) (unknown)	1,031	(1) Uptake of family planning<br>(2) Pregnancy outcomes
Kufa *et al.* 2018 [78]	Tuberculosis	People seeking HIV and/or TB testing or treatment services	South Africa (Ekurhuleni District)	2010-13	Cluster-randomised trial	Integration of HIV and TB services into primary healthcare clinics	Local public health clinic(s) (unknown)	4,182 (at 18 sites)	(1) Incidence of hospitalisation and deaths among newly diagnosed HIV patients<br>(2) Proportion of HIV patients newly diagnosed with TB
Lambdin *et al.* 2013 [80]	Primary healthcare	ART-naive PLHIV	Mozambique (Manica and Sofala Provinces)	2006-08	Retrospective cohort study	Integration of HIV care and treatment into primary healthcare	Local public health clinic(s) (urban, rural)	11,775 (at 17 sites)	(1) Attrition during early and late patient follow-up
Leon *et al.* 2010 [81]	SRH and STIs	People presenting with STI(s)	South Africa (Cape Town)	2007	Cluster-randomized controlled trial	Integration of provider-initiated (opt-out) HIV<br>testing and counselling into primary healthcare clinics mainly offering SRH services	Local public health clinic(s) (urban)	9,080 (at 21 sites)	(1) Uptake of HIV testing and counselling<br>(2) Consistency of testing across clinics
Van Lettow *et al.* 2014 [82]	Maternal and child healthcare	Newly identified HIV-positive women	Malawi	2012	Cohort study	Integration of ART and ANC into MCH clinics (stand-alone, fully integrated in one of the clinics or referrals from MCH clinic to ART clinic)	Multiple settings (urban, rural)	41,203 (at 141 sites)	(1) Proportion of women tested for HIV<br>(2) Retention rates
Mansoor *et al.* 2019 [84]	Family planning; PrEP	HIV-negative women	South Africa (KwaZulu-Natal)	2012-14	Randomized controlled trial	Integration of PrEP into family planning services (CAPRISA 004 trial)	Local public health clinic(s) (urban, rural)	372 (at 2 sites)	(1) Adherence rates<br>(2) Retention rates, number of returned used gel applicators<br>(3) HIV incidence rates
Mantell *et al.* 2017 [85]	SRH and STIs	People seeking SRH care	South Africa (Cape Town)	2006-11	Cluster-randomized controlled trial	Integration of SRH<br>into public sector HIV care services	Local public health clinic(s) (urban)	214 (at 1 site)	(1) Adherence to safer sex guidelines<br>(2) Use of contraceptives among HIV-positive clients
Matulionyte *et al.* 2019 [86]	Other infectious diseases	General population: hospitalized patients and outpatients	Lithuania (Vilnius)	2010-14	Retrospective cohort study	Integration of rapid HIV testing into infectious disease units at hopitals	Hospital(s) (urban)	4,911 (at 1 site)	(1) HIV prevalence<br>(2) Cost-effectiveness<br>(3) Clinical effectiveness<br>(4) Feasibility
Mavhu *et al.* 2020 [87]	SRH and STIs; mental health	HIV-positive adolescent girls and young women	Zimbabwe (Bindura and Shamva)	2016-17	Cluster-randomized controlled study	Integration of adherence support, SRH, and mental health services into ART programmes (Zvandiri project)	Local public health clinic(s) (urban, rural)	496 (at 16 sites)	(1) ART adherence<br>(2) Viral suppression

Mayhew *et al.* 2017 [88]	SRH and STIs; other infectious diseases	Users of SRH services	Kenya	2009-13	Non-randomised trial	Integration of HIV testing and counselling into SRH and MCH services (Integra Initiative)	Local public health clinic(s) (urban, rural)	8,841 (at 17 sites)	(1) Uptake of services<br>(2) Workload of healthcare staff<br>(3) Contextual factors influencing sustainability of integration
McBain *et al.* 2017 [89]	Primary healthcare	Newly enrolled HIV-positive patients	Malawi (Neno District)	2013-14	Population-based retrospective analysis	Integration of HIV care and surveillance into social, community, and nutritional support programs	Local public health clinic(s) (urban, rural)	129,938 (at 682 sites)	(1) Survival rate<br>(2) Cost for care<br>(3) QALYs gained
Mendelsohn *et al.* 2018 [90]	SRH and STIs; family planning	Adolescents visiting an integrated adolescent youth centre clinic or non-integrated public clinic	South Africa (Cape Town)	2015	Cohort study	Integration of sexual and reproductive health services into educational and recreational programmes	Local public health clinic(s) (urban)	2,235 (at 2 sites)	(1) SRH service uptake<br>(2) HIV-counselling and testing uptake<br>(3) Contraception use rates and adherence<br>(4) HIV-case detection rates
Mgbemena *et al.* 2015 [91]	Primary healthcare	PLHIV with chronic pain	USA	2012-13	Prospective cohort study	Integration of chronic pain relief therapy into a primary care HIV clinic setting	Local public health clinic(s) (unknown)	27 (at 1 site)	(1) Pain score reduction<br>(2) Costs
Miller *et al.* 2018 [92]	Treatment for substance use disorders; mental health	HIV-infected PWID (with ≥ 1 uninfected injection partner) and their partners	Ukraine, Indonesia, Vietnam	2015-18	Randomized controlled trial	Integration of systems navigation, psychosocial counselling, and CD4-independent ART-initiation into ART and opioid replacement therapy (HPTN 074 Vanguard Trial)	Community-level (urban)	502 (at multiple sites)	(1) Retention rates<br>(2) ART, opioid replacement therapy use rates, Viral suppression rates<br>(3) Mortality<br>(4) Injection Partner HIV incidence
Momplaisir *et al.* 2013 [93]	Primary healthcare; non-communicable diseases	HIV-positive and -negative patients seeking care in HIV-integrated, specialized HIV or general internal medicine facilities	USA (Pennsylvania)	2010-11	Cross-sectional study	Integration of colorectal and breast cancer screening into HIV- and general internal medicine clinics	Hospital(s) (urban)	762 (at 3 sites)	(1) Odds of getting cancer screening
Mudzengi *et al.* 2017 [95]	Tuberculosis	Adults living with TB and/or HIV	South Africa (Ekurhuleni)	2013	Cross-sectional study	Integration of TB-HIV services into public primary health care clinics (MERGE trial)	Local public health clinic(s) (urban, rural)	463 (at 18 sites)	(1) Costs of care
Musarandega *et al.* 2018 [96]	Maternal and child healthcare; PMTCT	Pregnant women	Zimbabwe (Hurungwe District)	2014-15	Pre-post study	Integration of provider-initiated HIV testing into infant health services	Local public health clinic(s) (urban, rural)	12,556 (at 33 sites)	(1) Women tested for HIV<br>(2) Infants tested for HIV<br>(3) Proportion of HIV-infected infants

Myer *et al.* 2018 [97]	Maternal and child healthcare; PMTCT	HIV-positive women eligible for ART in pregnancy, presenting with their infant(s) after delivery	South Africa (Cape Town)	2013-14	Randomized controlled trial	Integration of ART into postnatal MCH services	Local public health clinic(s) (urban)	471 (at 1 site)	(1) ART retention rate<br>(2) Viral suppression rate<br>(3) Transmission rate, child mortality, breastfeeding duration
Nance *et al.* 2017 [98]	Maternal and child healthcare	HIV-infected postpartum women and their children	Tanzania (Shinyanga Region)	2014-15	Cluster-randomized controlled trial	Integration of CHW-linkage, ART-adherence counselling, tracing and provision of birth planning tools into PMTCT facilities	Community-level (urban, rural)	1,830 (at 32 sites)	(1) Retention in care at 60, 120 days<br>(2) ART initiation rates, timing<br>(3) ART adherence rate
Ndagijimana *et al.* 2015 [99]	Tuberculosis	Communities from Kicukiro and Rulindo Districts	Rwanda (Kicukiro and Rulindo Districts)	2006-10	Cohort study	Integration of HIV and TB testing, treatment and care	Community-level (urban, rural)	1,695 (at 12 sites)	(1) Utility<br>(2) Quality<br>(3) TB health outcomes
Ngo *et al.* 2013 [100]	SRH and STIs; family planning	Adolescents (15-24) visiting integrated youth-friendly HIV/SRH services	Vietnam (five provinces)	2006-09	Pre-post study	Integration of HIV-testing, outreach activities, referral and counselling into SRH and family planning services (gynaecological check-ups, STI, family planning and ANC services)	Outpatient-clinic(s) (urban)	1,314 (at multiple sites)	(1) Testing rates<br>(2) Attitudes toward testing<br>(3) HIV knowledge and risk perceptions<br>(4) HIV-related risk behaviours
Nsubuga-Nyombi *et al.* 2019 [101]	Maternal and child healthcare; PMTCT	HIV-positive mothers and their HIV-exposed infants visiting one of 22 facilities	Uganda (Ninja, Kisoro, Manafwa, Namutumba, Ntungamo, and Tororo)	2013-15	Pre-post study	Integration of nutrition into PMTCT services	Local public health clinic(s) (urban, rural)	~2,000 (at 22 sites)	(1) Infant and young children feeding-counselling rate, adherence<br>(2) Service utilization rates<br>(3) HIV-exposed infants alive at 18 months, infection rate
Obure *et al.* 2015 [102]	SRH and STIs	Patients visiting one of 40 non-government and/or public health facilities	Kenya, eSwatini	2010-11	Non-randomised trial	Integration of HIV counselling, testing, treatment and care into family planning, ANC, PNC, cervical cancer screening and STI screening services (Integra Initiative)	Multiple settings (urban, rural)	11,471 (at 40 sites)	(1) Costs per visit
Obure, Sweeney *et al.* 2016 [103]	SRH and STIs	Patients visiting one of 40 non-government and/or public health facilities	Kenya, eSwatini	2008-09 & 2010-11	Non-randomised trial	Integration of HIV counselling, testing, treatment and care into family planning, ANC, PNC, cervical cancer screening and STI screening services (Integra Initiative)	Multiple settings (urban, rural)	11,471 (at 40 sites)	(1) Healthcare quality<br>(2) Technical efficiency of integration

Obure, Jacobs *et al.* 2016 [104]	SRH and STIs	Patients visiting one of 40 non-government and/or public health facilities	Kenya, eSwatini	2008-11	Non-randomised trial	Integration of HIV counselling, testing, treatment and care into family planning, ANC, PNC, cervical cancer screening and STI screening services (Integra Initiative)	Multiple settings (urban, rural)	11,471 (at 40 sites)	(1) Technical, allocative efficiency<br>(2) Service-specific economies of scale
Owiti *et al.* 2015 [106]	Tuberculosis	HIV and/or TB patients presenting at one of 17 rural public health facilities	Kenya (Western region)	2010-12	Pre-post cohort study	Integration of HIV into TB-services using three models: 1) one-stop-shop 2) HIV+TB and non-HIV TB clinics and 3) under-one-roof TB/HIV clinics with separate staff for each	Local public health clinic(s) (rural)	824 (at 17 sites)	(1) Uptake of ART<br>(2) Time to ART initiation<br>(3) TB treatment success among co-infected patients
Palma *et al.* 2018 [107]	Non-communicable diseases	PLHIV at risk of cardiovascular diseases	eSwatini (Manzini)	2015-16	Randomized controlled trial	Integration of cardiovascular disease screening into HIV clinic visits	Hospital(s) (urban)	172 (at 10 sites)	Impact of screening on:<br>(1) patient flow<br>(2) HIV service delivery
Rawat *et al.* 2018 [108]	Non-communicable diseases	PLHIV receiving ART in primary healthcare clinics	South Africa (Free State Province)	2009-13	Quasi-experimental study	Integration of HIV services into primary health care	Local public health clinic(s) (mixed)	57,958 (at 131 sites)	(1) Number of patients receiving ART<br>(2) Number of new diabetes and hypertension patients
Reza-Paul *et al.* 2019 [109]	SRH and STIs; HPV, cervical cancer	Female sex workers visiting a community-based clinic or partner private hospital	India (Mysore)	2013-16	Cohort study	Integration of SRH (STI and cervical cancer testing/treatment) and HIV services (testing/counselling) into general population SRH services (DIFFER study)	Community-level (urban)	873 (at multiple sites)	(1) SRH service uptake (condom use, STI and cervical cancer testing/treatment rates)<br>(2) Proportion of FSW accessing services<br>(3) HIV-testing rates
Rosen *et al.* 2021 [145]	Mental health	Adolescents living with HIV	Zambia (Central and Eastern Provinces)	2017	Prospective cohort study	Integration of psychosocial, economic strengthening, and clinical services to HIV- affected households for adolescents (Zambia Family (ZAMFAM) Project)	Community-level (mixed)	494 house-holds	(1) Self-reported health status<br>(2) HIV related stigma
Van Rie *et al.* 2014 [110]	Tuberculosis	TB patients	Democratic Republic of Congo (Kinshasa city)	2007-09	Prospective cohort study	Integration of nurse-centred TB and HIV services into a primary health clinic.	Local public health clinic(s) (unknown)	4,463 (at multiple sites)	(1) ART uptake/initiation<br>(2) CD4 cell counts<br>(3) TB-related mortality
Roberts *el al.* 2019 [111]	Family planning; Maternal and child healthcare; SRH and STIs; PrEP	Adolescent girls and young women visiting family planning and MCH clinics	Kenya (Western counties)	2017-18	Cross-sectional study	Integration of PrEP into family planning and mother-child health services (PrIYA programme, DREAMS Innovation Challenge)	Local public health clinic(s) (semi-urban)	24,005 (at 16 sites)	(1) Average cost per patient month of supplied PrEP using different protocols<br>(2) Unit costs and volume

Rodkjaer *et al.* 2017 [112]	Mental health	PLHIV: HIV patients with psychological challenges	Denmark (Aarhus)	N/A	Randomized controlled trial	Integration of a mental health intervention (mind-body approach) into care for HIV patients	Hospital(s) (urban)	29 (at 1 site)	(1) Feasibility<br>(2) Change of depression risk<br>(3) Level of coping self-efficacy, stress, and personal growth
Rosenberg *et al* 2010 [113]	Viral hepatitis; mental health	General population; PWID –<br>people from ethnic minorities attending mental health services	USA (Baltimore)	2006-08	Randomized controlled trial	Integration of screening/testing for HIV and hepatitis, immunization for hepatitis A and B, risk-reduction counselling, medical treatment referral and support into mental health services	Community-level (urban)	236 (at 4 sites)	(1) Cost per patient, breakdown<br>(2) Participation, testing and immunisation rates<br>(3) Referral<br>(4) Knowledge and reduction in risk behaviour
Rosenberg *et al.* 2018 [114]	SRH and STIs	Adolescent girls and young women	Malawi (Lilongwe)	2016-17	Cohort study	Integration of previously stand-alone HIV testing, family planning, STI syndromic management and condom provision plus youth-friendly provision, peer-educators and behavioural intervention – bi-directional (Girl Power Malawi)	Local public health clinic(s) (urban)	1,000 (at 4 sites)	Uptake of (1) HIV-testing, including pre- and post-test-counselling (2) condoms (3) hormonal contraception (4) dual method contraception (5) medical STI consultations
Rutaremwa *et al.* 2016 [115]	Family planning; SRH and STIs	Women (15+ years) using integrated or unintegrated HIV and SRH services during pregnancy and delivery of last child	Uganda	2011	Cross-sectional study	Integration of HIV into SRH and “women” services (data from USAID Demographic and Health Surveys)	Multiple settings (urban, rural)	9,691 (at multiple sites)	(1) Service utilization rates
Schulz *et al.* 2013 [117]	Tuberculosis	TB/HIV co-infected patients presenting at TB hospital	South Africa (Western Cape)	2009-11	Cohort study	Integration of ART into TB services and general HIV-care	Hospital(s) (urban)	271 (at multiple sites)	(1) TB treatment success<br>(2) ART treatment success (CD4 counts)
Shade *et al.* 2013 [118]	Family planning	Women attending HIV and/or family planning services	Kenya (Nyanza)		Cluster-randomized controlled trial	Integration of family planning into HIV care and treatment clinics	Local public health clinic(s) (unknown)	4,135 (at 18 sites)	(1) Costs<br>(2) Cost-efficiency<br>(3) Cost-effectiveness
Shade *et al.* 2020 [143]	NCDs	General population; PLHIV	Uganda	2015-16	Retrospective cohort study	Integration of hypertension screening into HIV clinics for HIV-positive and HIV-negative people (SEARCH)	Community-level (rural)	2,425 (at 10 sites)	(1) Additional costs

Shenoi *et al.* 2017 [119]	Tuberculosis	Population with high burden of HIV and TB	South Africa (Msinga, KwaZulu-Natal)	2010-12	Retrospective cohort study	Integration of TB intensive case finding into HIV intensive case finding and CD4 testing	Community-level (rural)	5,615 (at 322 sites)	(1) HIV testing uptake (2) CD4 cell counts (3) HIV yield (4) TB drug resistance
Shin *et al.* 2020 [146]	Maternal and child healthcare	Women, Children	India (Andhra Pradesh)	2014-16	2x2 factorial intervention trial	Integration of nutritional interventions for mother-child pairs living with HIV visiting HIV clinics	Community-level (rural)	600	(1) Body weight (2) CD4 counts
Siapka *et al.* 2017 [120]	Family planning	General population	Kenya	2009-11	Pre-post intervention study	Integration of HIV services into family planning counselling and provision (Integra Initiative)	Multiple settings (mixed)	3,713 (at 24 sites)	(1) Duration of consultation (2) Staff workload
Simeone *et al*. 2017 [121]	Treatment for substance use disorders	PLHIV with substance use disorders	USA (San Francisco)	2015	Cohort study	Integration of HIV-care-continuum patient monitoring and intervention-need recognition into opioid replacement therapy	Multiple settings (urban)	65 (3 sites)	(1) Retention rates (2) Viral suppression rates
Siregar *et al.* 2011 [122]	SRH and STIs	People accessing voluntary testing and counselling	Indonesia (Bandung)	2008-09	Cohort study	Integration of voluntary testing and counselling into hospital, STI and prison clinic services compared to HIV services offered at a community clinic	Multiple settings (urban)	1,954 (at 4 sites)	(1) Service utilisation (2) Costs (3) Clinical HIV outcomes
Solomon *et al*. 2019 [123]	Viral hepatitis; treatment for substance use disorders	PWID (and MSM)	India	2012-17	Cluster-randomised trial	Integration of HIV testing, prevention, and treatment into opioid replacement therapy and MSM health services	Other (urban)	12,726 (at 22 sites)	(1) Self-reported HIV testing (2) Exposure to HIV testing
Solomon *et al*. 2020 [124]	Viral hepatitis; treatment for substance use disorders	PWID; MSM	India	2013-16	Randomized controlled trial	Integration of HCV testing into HIV programs (HIV testing, preventive services and linkage to ART programmes)	Other (urban)	~11,721 (at 12 sites)	(1) Self-reported HCV testing (2) HCV antibody prevalence (3) HCV treatment initiation
Stockton *et al*. 2020 [144]	Mental health	PLHIV	Malawi	2017-18	Pre-post intervention study	Integration of depression screening into routine HIV care	Local public health clinic(s) (unknown)	501 (2 sites)	(1) ART initiation (2) Retention in care (3) Viral suppression
Sweeney *et al*. 2014 [125]	SRH and STIs	Patients visiting one of 40 non-government and/or public health facilities	Kenya, eSwatini	2010-11	Non-randomized trial	Integration of HIV counselling, testing, treatment and care into family planning, ANC, PNC, cervical cancer screening and STI screening services (Integra Initiative)	Multiple settings (urban, rural)	11,471 (at 40 sites)	(1) Staff workload (2) Efficiency of service delivery
Talama *et al.* 2020 [139]	HPV, cervical cancer	Women living with HIV	Malawi (Neno)	2016-18	Pre-post intervention study	Integration of cervical cancer screening into a dual HIV and non- communicable disease clinic	Local public health clinic(s) (rural)	749 (at 1 site)	(1) Detected number of women with cervical cancer (2) Number of women first time screened for cervical cancer

Tomlinson *et al.* 2014 [126]	Maternal and child healthcare; PMTCT	Pregnant women (17+ years) and their new-borns	South Africa (Umlazi)	2008-10	Randomized controlled trial	Integration of a standard care package (PMTCT, lactation counselling, new-born care and systems navigation) into home-based MCH services	Community-level (semi-urban)	4,137 (at 30 sites)	(1) Exclusive breastfeeding rate<br>(2) HIV-free survival at 12 weeks<br>(3) Service utilization<br>(4) Infant weight-for-length z-scores at 12 weeks
Topp *et al.* 2010 [128]	Primary healthcare	Outpatient department patients	Zambia (Lusaka)	2008-09	Cross-sectional study	Integration of ART into regular non-HIV outpatient department services	Outpatient-clinic(s) (urban)	~4,300 (at 2 sites)	(1) Acceptability<br>(2) Uptake and adherence<br>(3) Feasibility<br>(4) Resource allocation
Topp *et al.* 2013 [129]	Primary healthcare	Outpatient department patients	Zambia (Lusaka)	2008-11	Cross-sectional study	Integration of HIV services into outpatient department services in urban primary health care clinics	Outpatient-clinic(s) (urban)	~48,000 (at 12 sites)	(1) Resource and allocative efficiencies<br>(2) Organizational advantages
Tran, Jacobs *et al.* 2012 [130]	Treatment for substance use disorders	HIV-positive drug users	Vietnam	2009	Cohort study, mathematical modelling study	Integration of ART into methadone replacement therapy (same site or different site)	Other (urban)	370 (at multiple sites)	(1) Cost per QALY gained<br>(2) Incremental cost-effectiveness ratio
Tran, Houston *et al.* 2012 [131]	Treatment for substance use disorders	HIV-positive drug users	Vietnam	2009	Cohort study	Methadone maintenance therapy integrated into ART programmes	Other (urban)	370 (at multiple sites)	(1) Health-related quality of life at baseline, 3, 6 and 9 months<br>(2) Methadone therapy response Rates
Turan *et al.* 2015 [132]	Maternal and child healthcare; PMTCT	HIV-positive pregnant women accessing ANC services	Kenya	2009-11	Randomized controlled trial	Integration of PMTCT/ART into ANC clinics (SHAIP trial)	Local public health clinic(s) (rural)	1,172 (at 12 sites)	(1) Maternal HIV-care enrolment<br>(2) HAART initiation rates<br>(3) Three-month infant HIV-testing uptake
Uebel *et al.* 2013 [133]	Primary healthcare	Patients receiving ART counselling and/or treatment	South Africa (Free State Province)	2007-08	Cohort study during a randomized controlled trial study	Integration of ART, HIV testing/counselling, prevention services and monitoring into primary healthcare	Local public health clinic(s) (mixed)	9,252 (at ~200 sites)	(1) Risk of mortality<br>(2) Coverage<br>(3) Access
Vodicka *et al.* 2017 [134]	HPV, cervical cancer	Adult women attending clinic for cervical cancer screening	Kenya (Nairobi)	2014	Cross-sectional study, qualitative study	Integration of cervical cancer screening (different modalities) into HIV care clinics	Hospital(s) (urban)	148 (at 1 site)	(1) Marginal Costs per screening<br>(2) Cost components

Vodicka *et al.* 2019 [135]	HPV, cervical cancer	HIV-positive women with pre-cancerous cervical lesions	Kenya (Nairobi)	2014	Cross-sectional study	Integration of treatment of pre-cancerous cervical lesions into same-day HIV care	Hospital(s) (urban)	54 (at 1 site)	(1) Patient characteristics (2) Costs per procedure/treatments, savings
Wagner *et al.* 2021 [138]	Family planning	Serodiscordant couples	Uganda (Wakiso, Masaka, Mbale, Jinja, Rukugiri, Mbarara)	2017-19	Cluster-randomized controlled trial	Integration of family planning services into HIV clinics for serodiscordant couples (Our Choice)	Local public health clinic(s) (mixed)	189 (at 6 sites)	(1) Uptake of contraceptive methods (2) Costs
Wang *et al.* 2014 [136]	Maternal and child healthcare; PMTCT; viral hepatitis	Women visiting participating ANC clinics	China (multiple counties)	2010-13	Cluster-randomized trial	Integration of free tests for HIV, syphilis, hepatitis B and PMTCT into ANC clinics	Multiple settings (unknown)	4,529 (at 60 sites)	(1) Testing rates (2) Mother-to-child transmission rates
Wang *et al.* 2015 [137]	Maternal and child healthcare; other infectious diseases	Children below five years of age	Zambia (Southern Province)	2013	Cluster-randomized controlled trial	Integration of early infant HIV testing into an immunisation programme (diphtheria, pertussis, and tetanus vaccine) with or without operational assistance	Local public health clinic(s) (urban, rural)	~3,000 (at 60 sites)	(1) Vaccine doses distributed (2) HIV testing uptake (3) maternal re-testing for HIV
Washington *et al.* 2015 [37]	Maternal and child healthcare; PMTCT	HIV-positive women visiting ANC clinics	Kenya (Nyanza Province)	2009-12	Cluster-randomized controlled trial	Integration of HIV services (including PMTCT) into antenatal care care clinics (SHAIP trial)	Local public health clinic(s) (rural)	1,172 (at 12 sites)	(1) Retention rates (2) ART initiation rates, timing (3) Maternal and child health outcomes
Young *et al.* 2019 [38]	Maternal and child healthcare; SRH and STIs; other infectious diseases	Women seeking MCH services in a high-volume dispensary	Kenya (Western region)	2014-15	Non-randomized trial, mathematical modelling study	Integration of point-of-care-testing (HIV, syphilis, malaria, anaemia) into MCH dispensaries	Multiple settings (urban, rural)	183 (at 1 site)	(1) Nurse utilization (2) Waiting times
Zang *et al.* 2016 [39]	Primary healthcare	Adults seeking outpatient care and screened positive for HIV	China (Guangxi)	2014-15	Modelling study based on a cluster-RCT	Integration of rapid point-of-care HIV screening, CD4 and viral load testing, linkage to care and ART counselling/initiation into primary healthcare (CTN-0056 trial)	Outpatient-clinic(s) (urban)	478 (at 12 sites)	(1) QALY gained (2) Incremental cost-effectiveness ratio (3) ART access
Zulliger *et al.* 2014 [40]	SRH and STIs	Pregnant women attending ANC	South Africa (Cape Town)	2011-12	Cohort study	Integration of rapid initiation ART into standard ANC care and testing (Rapid initiation of ART in Pregnancy (RAP) programme)	Local public health clinic(s) (semi-urban)	190 (at multiple sites)	(1) Cost per patient (2) Cost per QALY saved, cost-effectiveness (3) Perinatal infections averted
Schackmann *et al.* 2011 [147]	Treatment for substance use disorders	HIV-positive patients with opioid-dependence	USA	2005-07	Cohort Study	Integration of HIV screening into opioid replacement therapy	Community-level (unknown)	352 (at 12 sites)	(1) # of monthly provider encounters (2) Median monthly clinic/treatment costs (3) Cost breakdown
