# Supplementary material for: Integrating HIV services and other health services: A systematic review and meta-analysis
Source: PLoS Med. 2021 Nov 9;18(11):e1003836. doi: 10.1371/journal.pmed.1003836 (PMC8577772; doi:10.1371/journal.pmed.1003836)
Supplement: S3 Table — (PDF) [file pmed.1003836.s007.pdf]

S3 Table. GRADE assessment of the quality of the included studies.

Author(s) and year of publication	Risk of bias	Inconsistency	Indirectness	Imprecision	Publication bias	Effect size	Quality	Justification
Akinleye *et al.* 2017 [34]	Likely (-1)	Undetected (0)	Undetected (0)	Undetected (0)	Undetected (0)	Large (+1)	Low	Cross-sectional study downgraded due to (1) likely risk of bias from small timeslot of intervention, upgraded due to large effect sizes and large sample size
Akker *et al.* 2012 [35]	Likely (-1)	Undetected (0)	Undetected (0)	Undetected (0)	Undetected (0)	Large (+1)	Low	Retrospective cohort study, downgraded due to risk of bias from type of date; extraction from medical records, upgraded due to large effect sizes
Aliyu *et al.* 2016 [50]	Undetected (0)	Undetected (0)	Undetected (0)	Undetected (0)	Undetected (0)	N/A	High	Cluster-randomised controlled trial, high level of evidence
Ameh *et al.* 2017 [61]	Likely (-1)	Undetected (0)	Undetected (0)	Likely (-1)	Undetected (0)	N/A	Low	Pre-post study downgraded due to (1) likely risk of bias from time and location differences (2) possible imprecision of evidence due to small sample sizes
Ansa *et al.* 2012 [72]	Likely (-1)	Undetected (0)	Likely (-1)	Undetected (0)	Undetected (0)	N/A	Low	Pre-post study downgraded due to (1) risk of bias from non-random sample and pre-post set-up and (2) indirectness due to difference in intervention (different levels of integration)
Bailey *et al.* 2017 [83]	Likely (-1)	Likely (-1)	Undetected (0)	Undetected (0)	Undetected (0)	N/A	Very low	Prospective cohort study downgraded due to (1) likely risk of bias from healthcare facility heterogeneity and (2) possible inconsistency due to included self-reported outcomes
Bergmann *et al.* 2017 [94]	Likely (-1)	Undetected (0)	Undetected (0)	Likely (-1)	Undetected (0)	N/A	Low	Pre-post study downgraded due to (1) likely risk of bias from time and location differences and (2) imprecision of evidence due to indirect cost estimation
Bindoria *et al.* 2014 [105]	Likely (-1)	Undetected (0)	Likely (-1)	Undetected (0)	Undetected (0)	Large (+1)	Moderate	Pre-post study downgraded due to (1) risk of bias from different settings and (2) possible indirectness of evidence due to indirect comparison, upgraded due to large affect sizes
Broughton *et al.* 2016 [116]	Likely (-1)	Undetected (0)	Undetected (0)	Likely (-1)	Undetected (0)	N/A	Low	Pre-post study downgraded due to (1) risk of bias from different settings (districts) and (2) possible imprecision of evidence due to small sample sizes
Brunie *et al.* 2016 [127]	Likely (-1)	Undetected (0)	Undetected (0)	Likely (-1)	Undetected (0)	N/A	Low	Pre-post study downgraded due to (1) risk of bias from different settings (districts) and (2) possible imprecision of evidence due to small sample sizes
Brunie *et al.* 2017 [36]	Likely (-1)	Undetected (0)	Undetected (0)	Likely (-1)	Undetected (0)	N/A	Low	Cluster-randomized controlled trial downgraded due to (1) likely risk of bias due to different geographical regions of intervention and control groups and (2) imprecision of evidence due to largely qualitative outcomes
Busza *et al.* 2019 [41]	Undetected (0)	Undetected (0)	Undetected (0)	Likely (-1)	Undetected (0)	N/A	Moderate	Randomised controlled trial downgraded due to likely imprecision of evidence due to qualitative nature of the outcomes
Carrico *et al.* 2019 [42]	Likely (-1)	Undetected (0)	Undetected (0)	Likely (-1)	Undetected (0)	N/A	Low	Randomised controlled trial downgraded due to (1) risk of bias from small sample size and (2) impression due to self-reported substance use
Chan *et al.* 2010 [43]	Undetected (0)	Undetected (0)	Undetected (0)	Undetected (0)	Undetected (0)	N/A	Low	Retrospective cohort study, low level of evidence

Chan *et al.* 2016 [44]	Likely (-1)	Undetected (0)	Undetected (0)	Undetected (0)	Undetected (0)	Large (+1)	Low	Retrospective cohort study downgraded due to risk of bias from use of non-randomised clinic record data, upgraded due to large sample sizes and effect sizes
Chen *et al.* 2020 [140]	Undetected (0)	Undetected (0)	Undetected (0)	Undetected (0)	Undetected (0)	N/A	Low	Cross-sectional study, low level of evidence
Church *et al.* 2015 [45]	Undetected (0)	Undetected (0)	Likely (-1)	Likely (-1)	Undetected (0)	N/A	Low	Non-randomised controlled trial downgraded due to (1) likely indirectness of evidence from difference in intervention (degree of integration) and (2) possible imprecision due to qualitative nature of observations
Ciampa et al. 2011 [46]	Undetected (0)	Undetected (0)	Undetected (0)	Undetected (0)	Undetected (0)	N/A	Low	Cohort study, low level of evidence
Click *et al.* 2012 [47]	Very likely (-2)	Undetected (0)	Undetected (0)	Undetected (0)	Undetected (0)	N/A	Low	Pre-post study downgraded due to very likely risk of bias from (1) time effects and facility heterogeneity and (2) incomplete data
Coleman *et al.* 2012 [48]	Likely (-1)	Undetected (0)	Undetected (0)	Undetected (0)	Undetected (0)	N/A	Very low	Retrospective cohort study downgraded due to (1) risk of bias from small number of responders and (2) likely inconsistency in controlling for relevant determinants
Conradie *et al.* 2013 [49]	Likely (-1)	Undetected (0)	Undetected (0)	Undetected (0)	Undetected (0)	Large (+1)	Low	Retrospective cohort study downgraded due to risk of bias from nonblinded study design (visitors of one integrated and one non-integrated clinic), upgraded due to large effect sizes
Criniti *et al.* 2011 [51]	Undetected (0)	Undetected (0)	Undetected (0)	Likely (-1)	Undetected (0)	N/A	Very low	Cohort study downgraded due to likely imprecision of outcome measures
Deo *et al.* 2012 [52]	Undetected (0)	Undetected (0)	Undetected (0)	Undetected (0)	Undetected (0)	N/A	Low	Prospective cohort study, remains low level of evidence
De la Flor *et al.* 2017 [79]	Likely (-1)	Undetected (0)	Likely (-1)	Undetected (0)	Undetected (0)	N/A	Low	Pre-post study downgraded due to (1) likely risk of bias from time effect and (2) incomplete comparison
Digre *et al.* 2021 [141]	Likely (-1)	Undetected (0)	Undetected (0)	Undetected (0)	Undetected (0)	N/A	Moderate	Pre-post study downgraded due to likely risk of bias from time effects and use of routinely collected data
Dovel *et al.* 2020 [142]	Undetected (0)	Undetected (0)	Undetected (0)	Undetected (0)	Undetected (0)	N/A	High	Cluster-randomised controlled trial, high level of evidence
Ezeanolue *et al.* 2015 [53]	Undetected (0)	Undetected (0)	Undetected (0)	Likely (-1)	Undetected (0)	N/A	Very low	Cohort study downgraded due to small sample size likely leading to imprecise evidence
Geelhoed *et al.* 2013 [54]	Undetected (0)	Undetected (0)	Undetected (0)	Likely (-1)	Undetected (0)	N/A	Moderate	Randomised controlled trial downgraded due to qualitative nature of part of the primary evidence
Gilbert *et al.* 2018 [55]	Undetected (0)	Likely (-1)	Undetected (0)	Undetected (0)	Undetected (0)	N/A	Moderate	Randomised controlled trial downgraded due partially self-reported outcomes
Golovaty *et al.* 2018 [56]	Undetected (0)	Undetected (0)	Undetected (0)	Undetected (0)	Undetected (0)	N/A	Low	Prospective cohort study, remains low level of evidence
Greig *et al.* 2012 [57]	Likely (-1)	Undetected (0)	Undetected (0)	Undetected (0)	Undetected (0)	N/A	Very low	Retrospective cohort study downgraded due to large heterogeneity of health facilities and implemented intervention

Guillaine *et al.* 2017 [58]	Undetected (0)	Undetected (0)	Undetected (0)	Undetected (0)	Undetected (0)	N/A	Low	Retrospective cohort study, low level of evidence
Hankin *et al.* 2016 [59]	Likely (-1)	Undetected (0)	Undetected (0)	Undetected (0)	Undetected (0)	N/A	Very low	Retrospective cohort study downgraded due to large heterogeneity of implemented intervention
Haraka *et al.* 2015 [60]	Undetected (0)	Undetected (0)	Likely (-1)	Undetected (0)	Undetected (0)	Large (+1)	Low	Retrospective cohort study downgraded due multiple interventions, upgraded due to large effect sizes
Harding *et al.* 2012 [62]	Undetected (0)	Undetected (0)	Undetected (0)	Undetected (0)	Undetected (0)	N/A	Low	Prospective cohort study, low level of evidence
Hemmer *et al.* 2015 [63]	Likely (-1)	Undetected (0)	Likely (-1)	Undetected (0)	Undetected (0)	N/A	Low	Pre-post study downgraded due to (1) likely risk of bias from time effects and (2) facility-level heterogeneity
Herce *et al.* 2018 [64]	Undetected (0)	Undetected (0)	Undetected (0)	Undetected (0)	Undetected (0)	N/A	High	Pre-post study, high level of evidence
Herlihy *et al.* 2015 [65]	Likely (-1)	Undetected (0)	Likely (-1)	Undetected (0)	Undetected (0)	N/A	Low	Pre-post study downgraded due to (1) likely risk of bias from time effects and facility-level heterogeneity and (2) multiple tested interventions
Hermans *et al.* 2012 [66]	Likely (-1)	Undetected (0)	Undetected (0)	Undetected (0)	Undetected (0)	N/A	Moderate	Pre-post study downgraded due to likely risk of bias from time effects and use of routinely collected data
Hewett *et al.* 2016 [67]	Undetected (0)	Undetected (0)	Undetected (0)	Undetected (0)	Undetected (0)	N/A	High	Randomised controlled trial, high level of evidence
Hung *et al.* 2016 [68]	Likely (-1)	Undetected (0)	Undetected (0)	Undetected (0)	Undetected (0)	N/A	Moderate	Pre-post study downgraded due to likely risk of bias from time effects and use of routinely collected data
Jacobson *et al.* 2015 [69]	Undetected (0)	Undetected (0)	Undetected (0)	Undetected (0)	Undetected (0)	N/A	Low	Retrospective cohort study, low level of evidence
Johns *et al.* 2017 [70]	Likely (-1)	Undetected (0)	Undetected (0)	Undetected (0)	Undetected (0)	N/A	Moderate	Pre-post study downgraded due to likely time effects and facility-level heterogeneity
Kanyuuru *et al.* 2015 [71]	Likely (-1)	Undetected (0)	Likely (-1)	Undetected (0)	Undetected (0)	Large (+1)	Moderate	Pre-post study downgraded due to (1) likely risk of bias from time and location differences and (2) indirectness of evidence because of intervention differences, upgraded due to large effect sizes.
Katz *et al.* 2016 [73]	Undetected (0)	Undetected (0)	Undetected (0)	Undetected (0)	Undetected (0)	N/A	Low	Cohort study, low level of evidence.
Kerschberger *et al.* 2012 [74]	Likely (-1)	Undetected (0)	Undetected (0)	Undetected (0)	Undetected (0)	N/A	Moderate	Pre-post study downgraded due to likely risk of bias from time effects and use of routinely collected data
Kimani *et al.* 2015 [75]	Likely (-1)	Undetected (0)	Undetected (0)	Undetected (0)	Undetected (0)	N/A	Moderate	Non-randomised trial downgraded due to possible risk of bias from participant' preferences for integrated *vs.* non-integrated health facility
Kinyua *et al.* 2019 [76]	Likely (-1)	Undetected (0)	Likely (-1)	Undetected (0)	Undetected (0)	Large (+1)	Moderate	Pre-post study downgraded due to (1) likely risk of bias from time effects and (2) facility-level heterogeneity, upgraded due to large effect sizes
Kosgei *et al.* 2011 [77]	Undetected (0)	Undetected (0)	Undetected (0)	Undetected (0)	Undetected (0)	N/A	Low	Retrospective cohort study, low level of evidence

Kufa *et al.* 2018 [78]	Undetected (0)	Undetected (0)	Likely (-1)	Undetected (0)	Undetected (0)	N/A	Moderate	Cluster-randomised trial downgraded due to facility-level heterogeneity
Lambdin *et al.* 2013 [80]	Undetected (0)	Undetected (0)	Undetected (0)	Undetected (0)	Undetected (0)	N/A	Low	Retrospective cohort study, low level of evidence
Leon *et al.* 2010 [81]	Undetected (0)	Undetected (0)	Likely (-1)	Undetected (0)	Undetected (0)	N/A	Moderate	Pre-post study downgraded due to likely heterogeneity between facilities and implementation of the intervention
Mansoor *et al.* 2019 [84]	Undetected (0)	Undetected (0)	Undetected (0)	Undetected (0)	Undetected (0)	N/A	High	Randomised controlled trial, high level of evidence
Mantell *et al.* 2017 [85]	Undetected (0)	Undetected (0)	Undetected (0)	Undetected (0)	Undetected (0)	N/A	High	Randomised controlled trial, high level of evidence
Matulionyte *et al.* 2019 [86]	Undetected (0)	Undetected (0)	Undetected (0)	Undetected (0)	Undetected (0)	N/A	Low	Retrospective cohort study, low level of evidence
Mavhu *et al.* 2020 [87]	Undetected (0)	Undetected (0)	Undetected (0)	Undetected (0)	Undetected (0)	N/A	High	Randomised controlled trial, high level of evidence
Mayhew *et al.* 2017 [188]	Likely (-1)	Undetected (0)	Undetected (0)	Undetected (0)	Undetected (0)	N/A	Moderate	Non-randomised trial downgraded due to likely risk of bias from different types of clinics in intervention and comparison groups
McBain *et al.* 2017 [89]	Undetected (0)	Undetected (0)	Undetected (0)	Undetected (0)	Undetected (0)	N/A	Low	Retrospective cohort study, low level of evidence
Mendelsohn *et al.* 2018 [90]	Likely (-1)	Undetected (0)	Undetected (0)	Undetected (0)	Undetected (0)	N/A	Low	Cohort study downgraded due to likely risk of bias from non-randomised comparison of two very different types of health facilities
Miller *et al.* 2018 [92]	Undetected (0)	Likely (-1)	Undetected (0)	Undetected (0)	Undetected (0)	N/A	Moderate	Randomised controlled trial downgraded due to self-reporting of outcomes by participants (ART use)
Momplaisir *et al.* 2013 [93]	Undetected (0)	Undetected (0)	Undetected (0)	Undetected (0)	Undetected (0)	N/A	Low	Cross-sectional study, low level of evidence
Mudzengi *et al.* 2017 [95]	Undetected (0)	Undetected (0)	Undetected (0)	Undetected (0)	Undetected (0)	N/A	Low	Cross-sectional study, low level of evidence
Musarandega *et al.* 2018 [96]	Likely (-1)	Undetected (0)	Likely (-1)	Undetected (0)	Undetected (0)	N/A	Low	Pre-post study downgraded due to (1) likely risk of bias from time differences and facility heterogeneity and (2) comparison of data from different sources
Myer *et al.* 2018 [97]	Undetected (0)	Undetected (0)	Undetected (0)	Undetected (0)	Undetected (0)	N/A	High	Randomised controlled trial, high level of evidence
Nance *et al.* 2017 [98]	Undetected (0)	Undetected (0)	Undetected (0)	Undetected (0)	Undetected (0)	N/A	High	Randomised controlled trial, high level of evidence
Ndagijimana *et al.* 2015 [99]	Likely (-1)	Undetected (0)	Undetected (0)	Undetected (0)	Undetected (0)	N/A	Very low	Cohort study downgraded due to (1) likely risk of bias from facility-level heterogeneity and (2) qualitative nature of the evidence
Ngo *et al.* 2013 [100]	Very likely (-2)	Undetected (0)	Undetected (0)	Undetected (0)	Undetected (0)	Large (+1)	Moderate	Pre-post study downgraded due to very likely risk of bias due to non-standardised sample groups from survey data, upgraded due to large effect size

Nsubuga-Nyombi *et al.* 2019 [101]	Likely (-1)	Undetected (0)	Likely (-1)	Undetected (0)	Undetected (0)	N/A	Low	Pre-post study downgraded due to (1) risk of bias from time differences and facility heterogeneity and (2) unclarity with regard to comparability of intervention
Obure *et al.* 2015 [102]	Likely (-1)	Undetected (0)	Undetected (0)	Likely (-1)	Undetected (0)	N/A	Low	Non-randomised trial downgraded due to (1) likely risk of bias from different types of clinics in intervention and comparison groups and (2) likely imprecision of estimated costs (conducted from health providers' perspective)
Obure, Jacobs *et al.* 2016 [104]	Likely (-1)	Undetected (0)	Undetected (0)	Undetected (0)	Undetected (0)	N/A	Moderate	Non-randomised trial downgraded due to likely risk of bias from different types of clinics in intervention and comparison groups
Obure, Sweeney *et al.* 2016 [103]	Likely (-1)	Undetected (0)	Undetected (0)	Likely (-1)	Undetected (0)	N/A	Low	Non-randomised trial downgraded due to (1) likely risk of bias from different types of clinics in intervention and comparison groups and (2) likely imprecision of estimated costs (conducted from health providers' perspective)
Owiti *et al.* 2015 [106]	Likely (-1)	Undetected (0)	Undetected (0)	Undetected (0)	Undetected (0)	N/A	Moderate	Pre-post study downgraded due to likely risk of bias from time differences and facility heterogeneity
Palma *et al.* 2018 [107]	Undetected (0)	Undetected (0)	Undetected (0)	Undetected (0)	Undetected (0)	N/A	High	Randomised controlled trial, high level of evidence
Rawat *et al.* 2018 [108]	Likely (-1)	Undetected (0)	Undetected (0)	Undetected (0)	Undetected (0)	N/A	Moderate	Pre-post study downgraded due to likely risk of bias from time effects and use of routinely collected data
Reza-Paul *et al.* 2019 [109]	Undetected (0)	Undetected (0)	Undetected (0)	Undetected (0)	Undetected (0)	Large (+1)	Moderate	Cross-sectional study, upgraded due to large effect size
Rosen *et al.* 2021 [145]	Undetected (0)	Undetected (0)	Undetected (0)	Undetected (0)	Undetected (0)	N/A	Low	Prospective cohort study, low level of evidence
Roberts *el al.* 2019 [111]	Undetected (0)	Undetected (0)	Undetected (0)	Likely (-1)	Undetected (0)	Large (+1)	Low	Cross-sectional study downgraded due to likely imprecision of measured costs, upgraded due to large effect size
Rodkjaer *et al.* 2017 [112]	Undetected (0)	Likely (-1)	Undetected (0)	Likely (-1)	Undetected (0)	N/A	Low	Randomised controlled trial downgraded due to (1) imprecision because of small sample size and (2) qualitative nature of some of the primary outcomes
Rosenberg *et al.* 2010 [113]	Undetected (0)	Undetected (0)	Undetected (0)	Undetected (0)	Undetected (0)	Large (+1)	Moderate	Cohort study upgraded due to nested study design within RCT and reported large effect sizes
Rosenberg *et al.* 2018 [114]	Undetected (0)	Undetected (0)	Undetected (0)	Undetected (0)	Undetected (0)	N/A	High	Randomised controlled trial, high level of evidence
Rutaremwa *et al.* 2016 [115]	Undetected (0)	Undetected (0)	Undetected (0)	Undetected (0)	Undetected (0)	N/A	Low	Cross-sectional study, low level of evidence
Schackmann *et al.* 2011 [147]	Undetected (0)	Undetected (0)	Undetected (0)	Undetected (0)	Undetected (0)	N/A	Low	Cohort study, low level of evidence
Schulz *et al.* 2013 [117]	Undetected (0)	Undetected (0)	Undetected (0)	Undetected (0)	Undetected (0)	N/A	Low	Cohort study, low level of evidence
Shade *et al.* 2013 [118]	Likely (-1)	Undetected (0)	Undetected (0)	Undetected (0)	Undetected (0)	N/A	Moderate	Cluster randomized controlled trial downgraded due to likely risk of bias from large facility heterogeneity

Shade *et al.* 2020 [143]	Undetected (0)	Undetected (0)	Undetected (0)	Undetected (0)	Undetected (0)	N/A	Low	Retrospective cohort study, low level of evidence
Shenoi *et al.* 2017 [119]	Undetected (0)	Undetected (0)	Undetected (0)	Undetected (0)	Undetected (0)	N/A	Low	Retrospective cohort study, low level of evidence
Shin *et al.* 2020 [146]	Undetected (0)	Undetected (0)	Likely (-1)	Undetected (0)	Undetected (0)	N/A	Moderate	Quasi-experimental study downgraded due to likely heterogeneity between facilities and implementation of the intervention
Siapka *et al.* 2017 [120]	Likely (-1)	Undetected (0)	Undetected (0)	Likely (-1)	Undetected (0)	N/A	Low	Pre-post study downgraded due to (1) likely risk of bias from facility differences and different time points and (2) wide confidence intervals
Simeone *et al.* 2017 [121]	Undetected (0)	Undetected (0)	Undetected (0)	Undetected (0)	Undetected (0)	N/A	Low	Cohort study, low level of evidence
Siregar *et al.* 2011 [122]	Undetected (0)	Undetected (0)	Undetected (0)	Undetected (0)	Undetected (0)	N/A	Low	Cohort study, low level of evidence
Solomon *et al.* 2019 [123]	Undetected (0)	Undetected (0)	Undetected (0)	Undetected (0)	Undetected (0)	N/A	High	Randomised controlled trial, high level of evidence
Solomon *et al.* 2020 [124]	Undetected (0)	Undetected (0)	Undetected (0)	Undetected (0)	Undetected (0)	N/A	High	Randomised controlled trial, high level of evidence
Stockton *et al.* 2020 [144]	Undetected (0)	Undetected (0)	Undetected (0)	Undetected (0)	Undetected (0)	N/A	High	Pre-post study, high level of evidence
Sweeney *et al.* 2014 [125]	Likely (-1)	Undetected (0)	Undetected (0)	Undetected (0)	Undetected (0)	N/A	Moderate	Non-randomised trial downgraded due to likely risk of bias from different types of clinics in intervention and comparison groups
Talama *et al.* 2020 [139]	Undetected (0)	Undetected (0)	Likely (-1)	Undetected (0)	Undetected (0)	N/A	Moderate	Pre-post study downgraded due to likely heterogeneity between facilities and implementation of the intervention
Tomlinson *et al.* 2014 [126]	Likely (-1)	Undetected (0)	Undetected (0)	Undetected (0)	Undetected (0)	N/A	High	Randomised controlled trial downgraded due to likely risk of bias from facility heterogeneity, upgraded due to large effect sizes and indication of confounders that diluted the effect of HIV-free survival
Topp *et al.* 2010 [128]	Undetected (0)	Undetected (0)	Undetected (0)	Undetected (0)	Undetected (0)	N/A	Low	Cross-sectional study, low level of evidence
Topp *et al.* 2013 [129]	Undetected (0)	Undetected (0)	Undetected (0)	Undetected (0)	Undetected (0)	N/A	Low	Cross-sectional study, low level of evidence
Tran, Jacobs *et al.* 2012 [130]	Undetected (0)	Undetected (0)	Undetected (0)	Likely (-1)	Undetected (0)	N/A	Very low	Cohort study downgraded due to likely imprecision of cost estimates because of large range of assumptions
Tran, Houston *et al.* 2012 [131]	Undetected (0)	Undetected (0)	Undetected (0)	Likely (-1)	Undetected (0)	N/A	Very low	Cohort study downgraded due to likely imprecision of cost estimates because of large range of assumptions
Turan *et al.* 2015 [132]	Undetected (0)	Undetected (0)	Undetected (0)	Undetected (0)	Undetected (0)	N/A	High	Randomised controlled trial, high level of evidence

Uebel *et al.* 2013 [133]	Undetected (0)	Likely (-1)	Undetected (0)	Undetected (0)	Undetected (0)	N/A	Moderate	Randomised controlled trial downgraded due to possible inconsistency in reported outcomes from different levels of integration as intervention and assessment if degree of integration by questionnaire
Van Lettow *et al.* 2014 [82]	Undetected (0)	Undetected (0)	Undetected (0)	Undetected (0)	Undetected (0)	N/A	Low	Prospective cohort study, low level of evidence
Van Rie *et al.* 2014 [110]	Undetected (0)	Undetected (0)	Undetected (0)	Undetected (0)	Undetected (0)	N/A	Low	Prospective cohort study, low level of evidence
Vodicka *et al.* 2017 [134]	Undetected (0)	Undetected (0)	Undetected (0)	Likely (-1)	Undetected (0)	Large (+1)	Low	Cross-sectional study downgraded due to likely imprecision of measured costs, upgraded due to large effect size
Vodicka *et al.* 2019 [135]	Undetected (0)	Undetected (0)	Likely (-1)	Likely (-1)	Undetected (0)	Large (+1)	Very low	Cross-sectional study downgraded due to large set of assumptions for modelling component (higher likeliness of indirectness and imprecision of the evidence), upgraded due to large effect size
Wagner *et al.* 2021 [138]	Undetected (0)	Undetected (0)	Likely (-1)	Undetected (0)	Undetected (0)	N/A	Moderate	Cluster-randomised trial downgraded due to facility-level heterogeneity
Wang *et al.* 2014 [136]	Likely (-1)	Undetected (0)	Undetected (0)	Undetected (0)	Undetected (0)	N/A	Moderate	Cluster-randomised controlled trial downgraded due to likely risk of bias from unblinded intervention and control groups
Wang *et al.* 2015 [137]	Undetected (0)	Undetected (0)	Undetected (0)	Likely (-1)	Undetected (0)	N/A	Moderate	Cluster-randomized controlled trial downgraded due to wide confidence intervals in service uptake
Washington *et al.* 2015 [37]	Undetected (0)	Undetected (0)	Undetected (0)	Undetected (0)	Undetected (0)	N/A	High	Randomised controlled trial, high level of evidence
Young *et al.* 2019 [38]	Likely (-1)	Undetected (0)	Undetected (0)	Undetected (0)	Undetected (0)	N/A	Moderate	Pre-post study downgraded due to likely risk of bias from time differences and facility heterogeneity
Zang *et al.* 2016 [39]	Undetected (0)	Likely (-1)	Undetected (0)	Undetected (0)	Undetected (0)	N/A	Moderate	Cluster-randomised controlled trial downgraded due to non-empirical nature of some used data sources
Zulliger *et al.* 2014 [40]	Undetected (0)	Undetected (0)	Undetected (0)	Undetected (0)	Undetected (0)	N/A	Low	Retrospective cohort study, low level of evidence
